# MultiSpaceNet: graph-based joint representation learning for paired spatial transcriptome–proteome data

**DOI:** 10.1093/bioinformatics/btag676

**Published:** 2026-09-25

**Authors:** Zhengqian Zhang, Binghong Chen, Jingyi Bai, Jialiang Wang, Junjun Ren, Chongyao Liu, Ziqi Liu, Yikun Cao, Yongzhuang Liu

**Affiliations:** School of Computer Science and Technology, Harbin Institute of Technology, Heilongjiang, 150001, China; School of Mathematics, Harbin Institute of Technology, Heilongjiang, 150001, China; School of Mathematics, Harbin Institute of Technology, Heilongjiang, 150001, China; School of Computer Science and Technology, Harbin Institute of Technology, Heilongjiang, 150001, China; School of Computer Science and Technology, Harbin Institute of Technology, Heilongjiang, 150001, China; The School of Future Technology, Harbin Institute of Technology, Heilongjiang, 150001, China; School of Mathematics, Harbin Institute of Technology, Heilongjiang, 150001, China; The School of Future Technology, Harbin Institute of Technology, Heilongjiang, 150001, China; School of Computer Science and Technology, Harbin Institute of Technology, Heilongjiang, 150001, China

## Abstract

**Motivation:**

Paired spatial assays now measure the transcriptome and the proteome on the same tissue section. Existing integration methods commit to a structural choice at the outset: a separate graph per modality, early fusion that folds the measured protein into the embedding so it can no longer be predicted, or intersection rules that discard most similarity edges. Each choice limits what one trained model can do afterwards.

**Results:**

We present MultiSpaceNet, a graph-based framework for joint representation learning from paired spatial transcriptomic and proteomic data. A section is represented as one cell graph over a shared node set, with spatial, transcriptomic and proteomic relations carried as typed edges and the two modality branches kept separate until a per-cell attention fusion. On five benchmark datasets, MultiSpaceNet outperforms nine published state-of-the-art methods in spatial domain identification (mean adjusted Rand index 0.472). Its leakage-free RNA-to-protein imputation matches or exceeds established methods for signal-bearing proteins, and it preserves biological structure across replicate sections better than all compared alternatives. A single trained model thus provides spatial domains, protein imputation, cross-section joint embedding and descriptive per-cell modality-dominance maps.

**Availability and implementation:**

Source code is available at https://github.com/yongzhuangliulab/MultiSpaceNet and archived at Zenodo (DOI 10.5281/zenodo.22667390 and 10.5281/zenodo.21488824). The scripts that regenerate the reported tables and figures, together with their machine-readable result summaries, are included in the repository (directory resubmission/) and its Zenodo archive.

## 1 Introduction

Spatial transcriptomics profiles gene expression together with spatial position in tissue, so the relative arrangement of cells can be read out directly—information, i.e. central to disease pathology. Popular experimental methods include 10× Visium (Ji *et al.* 2020), Slide-seq (Rodriques *et al.* 2019, Stickels *et al.* 2021), and HDST (Vickovic *et al.* 2019). The resulting maps help characterize cellular functions and the interactive biological networks that underlie them.

Spatial proteomics resolves protein localization, abundance and interactions in situ (Lundberg and Borner 2019), and falls into two broad families. Mass-spectrometry-based methods, such as deep visual proteomics (Mund *et al.* 2022), couple laser microdissection with MS to profile regions of interest while preserving spatial context. Multiplexed imaging methods instead detect dozens of proteins by iterative staining or metal-ion labelling, as in CODEX (Goltsev *et al.* 2018) and imaging mass cytometry (Giesen *et al.* 2014). Together these have advanced the study of subcellular protein localization, cell–cell interactions and the tumour microenvironment (Keren *et al.* 2018, Schürch *et al.* 2020).

Spatial multi-omics technologies capture several modalities on a single section while preserving spatial context. Sequencing-based assays use barcoding and co-assay strategies to profile transcriptome and protein together: SPOTS (Ben-Chetrit *et al.* 2023) and spatial CITE-seq (Liu *et al.* 2023b) pair transcriptomics with DNA-barcoded antibodies, Stereo-CITE-seq (Liao *et al.* 2023) adds subcellular resolution, and related designs extend to chromatin accessibility (Zhang *et al.* 2023) or use alternative barcoding chemistries (Liu *et al.* 2020, Vickovic *et al.* 2022). Imaging-based platforms such as MERSCOPE (Liu *et al.* 2023a) and CosMx (He *et al.* 2022) instead read out transcripts, and increasingly protein markers, in situ at high resolution. Paired RNA–protein assays of this kind are the setting we target: each cell carries two measurements whose relationships are complementary rather than redundant.

Integrating heterogeneous spatial multi-omics data into a unified tissue map requires accounting for both their complementary information and spatial relationships. Existing multi-omics integration methods can be broadly categorized into non-spatial and spatial approaches. Non-spatial methods such as Seurat WNN (Hao *et al.* 2021), MOFA+ (Argelaguet *et al.* 2020), StabMap (Ghazanfar *et al.* 2024), totalVI (Gayoso *et al.* 2021), MultiVI (Ashuach *et al.* 2023), and scMM (Minoura *et al.* 2021) employ factor models, joint latent spaces, variational inference, or multi-view representation learning. Spatial tools such as STAGATE (Dong and Zhang 2022), SpaGCN (Hu *et al.* 2021), and GraphST (Long *et al.* 2023) build graph-based representations for a single modality, while SpatialGlue (Long *et al.* 2024) uses dual graphs for spatial multi-omics. Generative models have also been applied to adjacent single-modality problems, such as denoising spatial transcriptomics (Jiao *et al.* 2026) and extending gene-panel coverage in imaging-based assays (Wang *et al.* 2026a). Recent paired or mosaic spatial multi-omics methods include SpaMI (Gao *et al.* 2025), SMART (Du *et al.* 2026), SpaMosaic (Yan *et al.* 2026), CoMo (Gui *et al.* 2026) and SpatialCOC (Li *et al.* 2026a), spanning contrastive fusion, graph metric learning, mosaic integration and continuous spatial cross-omics correction. HEIST (Madhu *et al.* 2026) instead develops a pretrained graph foundation model for spatial transcriptomics and proteomics tasks. These methods make clear that the relevant question is no longer whether spatial graphs and modality fusion can be combined, but whether a particular paired-data formulation is useful under matched evaluation and where its boundaries lie.

Two methods published in July 2026 are especially close to our setting. SpaMFG (Li *et al.* 2026b) uses spatially weighted feature grouping and grouped factor integration. ARISE (Wang *et al.* 2026b) intersects RNA-similarity and spatial-proximity graphs to form an RNA-anchored shared topology, then fuses auxiliary modalities hierarchically. ARISE is therefore the most direct contemporary comparator for topology design: its shared-edge scaffold contrasts with our union of three typed relations, and the contrast is consequential—intersecting relations improves edge precision but, at the cell densities of these assays, discards most of the similarity graph, whereas a typed union preserves every relation’s full coverage (Results, “Spatial domain identification”). We reproduce both releases.

We propose MultiSpaceNet, a graph-based framework for joint representation learning from paired spatial transcriptome–proteome data. A section is represented as a single cell graph carrying three relations—spatial proximity, RNA feature similarity and protein feature similarity—over one shared node set; HANConv-based attention aggregates across them, a per-cell module fuses the two modalities, a SAGEConv layer refines along spatial edges, and the whole is trained by self-supervised reconstruction and cross-modal prediction. The contribution is stated in the terms this literature uses: the formulation, the read-outs it makes possible, and measured performance under a protocol fixed in advance. From one trained model, four read-outs follow: spatial domains; leakage-free RNA-to-protein imputation from the modality-separable RNA branch—a read-out that an early-fusion joint embedding of the same paired section cannot provide, because that embedding has already consumed the protein it would be asked to predict; a joint embedding of replicate sections; and per-cell modality-dominance maps, reported as a descriptive interpretability read-out. All comparisons run under the fixed protocol, with model-free references and matched untrained and no-graph counterfactuals in the main tables, so a reader can see what the formulation itself contributes.

Three results support this. First, on spatial domain identification across five datasets spanning three platforms (SPOTS, Stereo-CITE-seq, 10 × Visium + CITE-seq) and two species, under a single unified clustering protocol and a training budget fixed in advance, MultiSpaceNet ranks first against nine published baselines evaluated on all five datasets—including the topology-focused ARISE—and against SpatialCOC on the four shared mouse datasets supported by its official preprocessing. Second, because any such ranking could reflect the architecture acting as a fixed non-linear feature map rather than anything learned, we score the identical network with the training loop skipped: training raises domain ARI and retention of the limiting modality’s neighbourhood structure on all five datasets. Third, across replicate sections message passing preserves biological structure better than a matched model without it, although dedicated integrators achieve stronger batch removal, so we state the operating boundary explicitly and do not present MultiSpaceNet as a batch-correction tool.

SpaMFG is reproduced from its deterministic release on all five datasets: the common evaluator succeeds on three, while its released native clustering is reported on all five and is never substituted into the common-score table. ARISE is run for five seeds using its official preprocessing, graph builder, model, loss and fixed 350-epoch schedule. Because the released helper selects the best epoch using ground-truth ARI, our benchmark instead scores the final epoch without consulting labels during optimization.

We are equally explicit about what is not established. The framework assembles established graph operators rather than proposing a new one. The evidence does not show that relation typing, or any individual component, is necessary; the ablations that bound this are reported in full. The per-cell attention yields spatially structured modality-dominance maps, but a local-corruption stress test shows that the weights do not reliably identify or downweight a damaged branch, so the maps are descriptive attributions rather than causal or quality-calibrated explanations. Keeping one encoder per modality leaves the RNA representation modality-separable and supports leakage-free cross-modal protein imputation; we report that as a practical within-section route where protein was not measured rather than as evidence about representation quality.

## 2 Methods

### 2.1 Overview

MultiSpaceNet is organized as a three-panel architecture (Fig. 1): (i) *Input and feature encoding*—modality-specific MLP encoders transform RNA and protein inputs into latent vectors, which are fused by a cell-level attention module; (ii) *Heterogeneous graph learning*—a multi-relational cell graph with three edge types is processed by stacked HANConv and SAGEConv layers to yield the final embedding z; (iii) *Self-supervised decoding*—three decoders (RNA reconstruction, protein reconstruction, and cross-modal protein prediction from pre-fusion RNA features) drive end-to-end training; the learned z is used for downstream spatial domain identification, and the modality-separable pre-fusion embedding $h_{\text{rna}}$ provides the route to protein imputation. The model learns a joint cell embedding $z\in R^{N\times d}$ from RNA features $X_{\text{rna}}\in R^{N\times D_{r}}$ and protein features $X_{\text{prot}}\in R^{N\times D_{p}}$ together with spatial coordinates $S\in R^{N\times 2}$, where *N* is the number of cells/spots.

**Figure 1:**
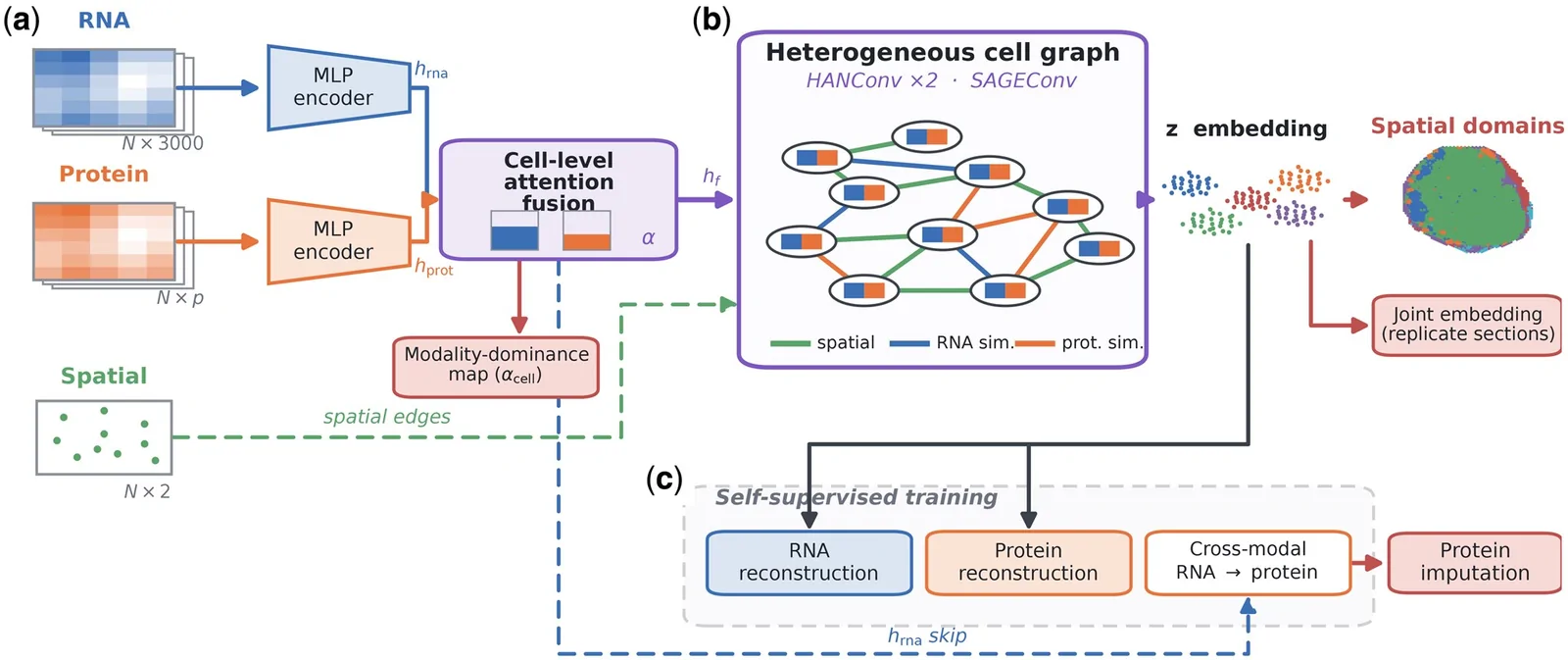
Overview of the MultiSpaceNet architecture. (a) *Input and encoding*. RNA (PCA-reduced), protein (CLR-normalized) and spatial coordinates are taken from one paired section. Modality-specific two-layer MLP encoders give $h_{\text{rna}}$ and $h_{\text{prot}}$, fused by cell-level attention into $h_{\text{fused}}$; coordinates are used only for graph construction (Methods). (b) *Heterogeneous graph learning*. One graph with three edge types—spatial proximity, RNA similarity and protein similarity—over a shared node set. Two HANConv layers aggregate across edge types, a SAGEConv layer propagates along spatial edges, and an MLP projects to the latent representation z. (c) *Self-supervised decoding*. RNA and protein reconstruction decoders from z, and a cross-modal decoder from $h_{\text{rna}}$, are trained jointly (Methods, Equation 6). The four read-outs of the one trained model are shown in red: spatial domains from z, the joint embedding of replicate sections from the same z, the per-cell modality-dominance map from $\alpha_{\text{cell}}$, and the protein-imputation route from the modality-separable $h_{\text{rna}}$ (Results, “Cross-modal protein expression prediction”).

### 2.2 Data preprocessing

#### 2.2.1 RNA preprocessing

Following the standard Scanpy (Wolf *et al.* 2018) pipeline, we filter genes expressed in fewer than 10 cells, select 3000 highly variable genes (HVGs) using the Seurat v3 flavour, normalize to 10 000 counts per cell, and apply log1p transformation. Optional scaling is applied in a platform-specific manner. PCA reduces the dimensionality to $D_{r}$ components.

#### 2.2.2 Protein preprocessing

We apply centred log-ratio (CLR) normalization following the Seurat convention, optionally scale, and apply PCA to $D_{p}$ components.

#### 2.2.3 Platform-specific processing

For SPOTS data (mouse spleen), we apply scaling and use $k_{s}=3$ spatial neighbours. For Stereo-CITE-seq data (mouse thymus), we skip scaling and filter cells with fewer than 80 expressed genes, as well as proteins detected in fewer than 50 cells.

### 2.3 Heterogeneous graph construction

We construct a heterogeneous graph G=(V,E) with a single node type (*cell*) and **three** edge types, each encoding a distinct view of cell–cell relationships:

1. *Spatial edges* $E_{s}$: *k*-nearest neighbours based on Euclidean distance in spatial coordinates S, capturing physical tissue proximity.
2. *RNA similarity edges* $E_{r}$: *k*-nearest neighbours based on correlation distance in RNA PCA space, capturing transcriptomic similarity.
3. *Protein similarity edges* $E_{p}$: *k*-nearest neighbours based on correlation distance in protein PCA space, capturing proteomic similarity.

All edge sets are symmetrized to ensure bidirectional message passing. The spatial graph uses $k_{s}\in \{3,6\}$ neighbours (platform-dependent), while the feature similarity graphs use $k_{f}=20$ neighbours with correlation distance.

The three relations are intended to express three complementary notions of proximity—spatial neighbours share a tissue microenvironment, RNA-similar cells share transcriptomic programs, and protein-similar cells share functional states—so that a paired section can be handled in one graph rather than by choosing between them. This is the motivation for the formulation; whether the separation into distinct relation types is individually necessary is tested in the ablations (Supplementary Tables S7 and S19), and it is not established there.

### 2.4 Modality-specific encoders

Each modality m∈{rna,prot} is processed by an independent two-layer residual MLP encoder:


(1) $$h_{m}=\text{ELU}(\text{BN}(W_{2}^{m}\text{Dropout}(\text{ELU}(\text{BN}(W_{1}^{m}X_{m}))))+W_{\text{res}}^{m}X_{m}),$$

where BN denotes batch normalization and $W_{\text{res}}^{m}$ is a linear residual projection used when the input dimension differs from the hidden dimension *d*. The residual connection ensures stable gradient flow and preserves input information.

### 2.5 Cell-level attention fusion

Rather than using a fixed global weighting scheme, we learn per-cell attention weights over the two modalities. Given the encoded representations $h_{\text{rna},i}$ and $h_{\text{prot},i}$ for cell *i*, we compute:


(2) $$\alpha_{i}=\text{softmax}(\text{MLP}([h_{\text{rna},i};\;h_{\text{prot},i}]))\;\in \;R^{2},$$

(3) $$h_{\text{fused},i}=\alpha_{i,1}h_{\text{rna},i}+\alpha_{i,2}h_{\text{prot},i},$$

where [·;·] denotes concatenation and the MLP consists of two linear layers with ELU activation. This mechanism allows different cells to assign different importance to RNA versus protein modalities based on their individual molecular profiles.

### 2.6 Graph convolution and output

The fused representations are propagated by L=2 layers of heterogeneous attention network convolution (HANConv) (Wang *et al.* 2019), which learns node-level attention within each edge type (H=4 heads) together with a semantic-level weight $\beta_{\phi }$ over the three types; each layer is followed by a residual connection, layer normalization, ELU and dropout. A single GraphSAGE layer (SAGEConv) then refines the representation along spatial edges only, and a linear projection maps it to the final embedding $z\in R^{N\times d}$. The layer equations and the cosine learning-rate schedule are given in Supplementary “Model architecture and optimization details”.

### 2.7 Self-supervised training objectives

The model is trained entirely in a *self-supervised* fashion without requiring any cell-type labels. Three loss terms are combined:

#### 2.7.1 Reconstruction loss

We reconstruct the original modality features from the joint embedding z via two independent 2-layer MLP decoders:


(4) $$L_{\text{recon}}=w_{r}\|X_{\text{rna}}-{\hat{X}}_{\text{rna}}{\|}_{2}^{2}+w_{p}\|X_{\text{prot}}-{\hat{X}}_{\text{prot}}{\|}_{2}^{2},$$

where ${\hat{X}}_{m}={\text{Decoder}}_{m}(z)$.

#### 2.7.2 Cross-modal prediction loss

We predict protein features directly from the *pre-fusion* RNA encoder output $h_{\text{rna}}$ using a 3-layer MLP decoder $f_{\text{cross}}$:


(5) $$L_{\text{cross}}=w_{c}\|X_{\text{prot}}-f_{\;\text{cross}}(h_{\text{rna}}){\|}_{2}^{2}.$$

This term supplies a protein-supervised gradient to the RNA branch while keeping $h_{\text{rna}}$ modality-separable, which is what makes the leakage-free imputation read-out available at inference. Because the 128-dimensional pre-fusion embedding is a non-linear expansion of the RNA principal components, an expressive downstream predictor can benefit without the joint representation itself being superior; we therefore report imputation as a practical read-out rather than evidence of integration quality.

#### 2.7.3 Total loss

The overall training objective is the sum of the above terms:


(6) $$L=L_{\text{recon}}+L_{\text{cross}}.$$

The weights $w_{r}$, $w_{p}$, and $w_{c}$ control the relative contribution of each component and are fixed to a single dataset-independent setting ($w_{r}=1,w_{p}=3,w_{c}=1$) under the *a-priori* protocol (Implementation details; Table S1).

### 2.8 Evaluation pipeline

Following the SpatialGlue evaluation protocol, we evaluate spatial domain identification through the following steps:

1.
$L_{2}$ -normalize the embedding z;
2. if the embedding has more than 20 dimensions, reduce it to 20 via PCA; otherwise retain its existing dimensionality;
3. apply Gaussian model-based clustering using mclust (Scrucca *et al*. 2016) with the EEE model (equal volume, equal shape, equal orientation) and a fixed random seed of 2020;
4. compute the Adjusted Rand Index (ARI) against ground-truth annotations.

This identical pipeline—including setting the number of clusters *K* to the ground-truth spatial-domain count for every dataset (5/5/8/6/6) and method—is applied to all baselines, so that differences in ARI reflect the learned embeddings rather than differences in clustering or cluster-number selection. Here “identical” refers to the embedding-to-ARI scoring stage: every method produces its embedding through its *own* published preprocessing and model, and we then score all embeddings with the same clustering pipeline and *K*. The ARI uses the annotated cells returned by that native pipeline; retained-cell counts are stored with every per-seed result, so any method-native cell filtering is auditable rather than silently imputed. The multi-omics methods consume the paired RNA and protein modalities directly, whereas the single-omics spatial baselines (STAGATE, GraphST, SpaGCN) are given the concatenated RNA+protein feature matrix, the standard way to apply a single-omics method to paired data. Per-method settings and hyperparameter provenance are listed in Table S13.

### 2.9 Implementation details

#### 2.9.1 Architecture and training (fully *a-priori* protocol)

The backbone is as specified in Methods: 2-layer MLP encoders into a shared 128-dimensional space, per-cell attention fusion, two 4-head HANConv layers, one SAGEConv layer for spatial refinement, and a linear output projection, with batch normalization, dropout (*P* = .1) and ELU between layers. Training uses Adam ($\text{lr}=10^{-3}$, CosineAnnealingLR to $\eta_{\text{min}}=10^{-5}$). To avoid selecting hyperparameters on the evaluation metric, all five datasets use a *single, dataset-independent* configuration (Table S1): a fixed budget of 1000 epochs (cosine $T_{\text{max}}=1,000$), fixed loss weights ($w_{r}=1$, $w_{p}=3$, $w_{c}=1$), and $k_{f}=20$ feature-similarity neighbours, with only the spatial neighbour count set by platform convention ($k_{s}=3$ for SPOTS, 6 otherwise). The budget was chosen by unsupervised training-loss convergence, *not* by ARI: on every dataset the self-supervised loss is within 1% of its final value by epoch 816–843 and changes by less than 0.7% over the final 100 epochs (Fig. S6). Compared with per-dataset epoch and loss-weight selection, the ARIs change by at most 0.05, so the gains are not a tuning artefact.

#### 2.9.2 Adapting to new data

The configuration is intended to be used as-is. Only $k_{s}$ and $w_{p}$ carry any dataset dependence, and both follow simple conventions rather than metric tuning: $k_{s}$ reflects array geometry, and $w_{p}$ balances the two reconstruction terms. Both modalities enter the encoders as principal components of the same dimension, so the imbalance $w_{p}$ corrects is one of scale rather than of dimensionality: across these datasets the RNA block carries 5.6–47.7× the total variance of the protein block, so an unweighted objective is dominated by RNA. We set $w_{p}=3$  *a priori* for every dataset. We report the sensitivity rather than only the choice: the structure-preservation balance is monotone in $w_{p}$, and on spatial-domain ARI the difference between $w_{p}=1$ and $w_{p}=3$ is +0.011 over 10 seeds with $w_{p}=1$ ahead on two of five datasets, so the setting is not critical on that axis (Table S21). For a panel whose variance is closer to that of the RNA block, $w_{p}$ nearer 1 is a reasonable starting point (Supplementary “Recommended defaults and adapting to new data”).

#### 2.9.3 Evaluation and environment

Spatial-domain identification is scored identically for every method through the unified embedding-to-clustering pipeline specified once in Methods, “Evaluation pipeline.” MultiSpaceNet results use 10 random seeds (mean ± SD). Training takes 1–2 minutes per dataset on one NVIDIA RTX 4090 (Python 3.9, PyTorch Geometric 2.6.1).

## 3 Results

### 3.1 Datasets

We evaluate MultiSpaceNet on five spatial multi-omics datasets spanning three sequencing platforms and two species (Table 1).

**Table 1:** Summary of the five spatial multi-omics datasets.

Dataset	Platform	Tissue	Spots	HVGs	Proteins	Clusters
Spleen Rep1	SPOTS	Mouse spleen	2568	3000	21	5
Spleen Rep2	SPOTS	Mouse spleen	2768	3000	21	5
Thymus Rep1	Stereo-CITE-seq	Mouse thymus	4469	3000	51	8
Thymus Rep2	Stereo-CITE-seq	Mouse thymus	4183	3000	19	6
Lymph Node A1	10× Visium CITE-seq	Human lymph node	3484	3000	31	6

HVGs, number of highly variable genes retained; both modalities are PCA-reduced before the modality encoders (Methods). Proteins: antibody-panel size.

#### 3.1.1 Datasets

The mouse spleen replicates (Ben-Chetrit *et al.* 2023) were acquired with SPOTS, which couples 10× Visium transcriptomics to DNA-barcoded antibody-derived tags (ADT) on one section, and carry five annotated compartments including white pulp, red pulp, and marginal zone. The mouse thymus replicates were generated with Stereo-CITE-seq (Liao *et al.* 2023), giving subcellular-resolution transcriptomics with modest, non-overlapping antibody panels (51 and 19 markers) across cortex, medulla and specialized regions; they test the model in an RNA-dominant regime. The human lymph node (Long *et al.* 2024) was processed from formalin-fixed paraffin-embedded (FFPE) tissue on the CytAssist Visium platform with paired 31-antibody CITE-seq, and adds a second species and platform. Per-dataset sizes, panel sizes and domain counts are given in Table 1.

### 3.2 Spatial domain identification

We evaluate spatial domain identification under one unified embedding-to-clustering protocol, specified once in Methods, “Evaluation pipeline” (L2 → PCA capped at 20 dimensions → mclust EEE, seed 2020). Nine baselines are evaluated on all five datasets: the spatial multi-omics methods SpatialGlue (Long *et al.* 2024), SMART (Du *et al.* 2026), SpaMI (Gao *et al.* 2025), SpaMosaic (Yan *et al.* 2026), CoMo (Gui *et al.* 2026) and ARISE (Wang *et al.* 2026b), and the established single-omics spatial methods GraphST (Long *et al.* 2023), SpaGCN (Hu *et al.* 2021) and STAGATE (Dong and Zhang 2022), which receive concatenated RNA+protein features. SMART, SpaMI and SpaMosaic were run from their public code repositories (https://github.com/Xubin-s-Lab/SMART-main, https://github.com/Gaocongqiang/SpaMI and https://github.com/JinmiaoChenLab/SpaMosaic); per-method settings are summarized in Table S13. ARISE is a particularly relevant topology comparator because it uses the intersection of RNA-similarity and spatial-proximity graphs as a shared scaffold for auxiliary modalities, whereas MultiSpaceNet retains three typed relations. SpatialCOC (Li *et al.* 2026a) is additionally evaluated on the four shared mouse datasets accepted by its official preprocessing; the unsupported 10× Visium CITE-seq lymph-node entry is left blank rather than supplied by an unpublished adaptation.

We also reproduce SpaMFG (Li *et al.* 2026b), published in July 2026, from its released deterministic script on all five datasets. Its common-evaluator score is available on three; on both thymus embeddings mclust EEE returns no fitted model, so those entries remain blank and the released Leiden result is reported separately (Table S14) rather than substituted. For ARISE, the official training helper evaluates ground-truth ARI at every epoch and returns the best-ARI embedding. To keep the comparison label-free during optimization, we preserve its official 350-epoch schedule and hyperparameters but score the final-epoch embedding; labels are loaded only for the downstream evaluator.

MultiSpaceNet ranks first on all five datasets (Table 2): under fully *a-priori* training it improves over SpatialGlue by +15% to +92% (paired Wilcoxon *P* ≤ .014; Table S2). Consistent with our treatment of the bimodal Lymph Node below, the upper endpoint is computed from that dataset’s median rather than its mean; on the mean it would read +137%. It also exceeds every additional published comparator on the datasets each supports. The largest absolute scores are on Thymus Rep2 (0.688) and the Human Lymph Node (0.570). ARISE obtains 0.357, 0.215, 0.217, 0.239 and 0.395 under the label-free final-epoch protocol, so the ranking also holds against the closest topology-focused comparator (on Spleen Rep1, 0.372 ± 0.016 against 0.357 ± 0.077, the two are within seed noise and the ordering is a point estimate). The mechanism behind this gap is measurable. ARISE’s intersection rule requires an RNA-similarity edge to coincide with a spatial edge, trading edge coverage for edge precision, and at these cell densities coverage binds: under ARISE’s own preprocessing and default neighbourhood size, the intersection retains 1.1%–2.7% of the similarity edges and leaves 71.6%–83.8% of cells with no intersection edge on the five datasets, and that intersection graph is the only neighbourhood ARISE’s protein branch aggregates over. A typed union keeps every relation’s full edge set, so no modality’s message passing is starved by a coincidence requirement between relations; this is a statement about which edges exist under each construction rule, not a claim that relation typing is necessary (the measurement script is included in the code repository). Per-method protocols and hyperparameter provenance are summarized in Table S13. Native-protocol checks for SMART, SpaMI, SpaMFG and ARISE retain the ranking on all available datasets; for SpaMFG they also document the two common-evaluator failures rather than masking them (Table S14).

**Table 2:** Spatial domain identification performance (ARI) across all five datasets.

Method	Spleen Rep1	Spleen Rep2	Thymus Rep1	Thymus Rep2	Lymph node
**MultiSpaceNet (Ours)**	0.372 ± 0.016	0.253 ± 0.031	0.475 ± 0.025	0.688 ± 0.092	0.570 ± 0.214
Spatial multi-omics methods					
SpatialGlue	0.323 ± 0.053	0.152 ± 0.023	0.361 ± 0.031	0.501 ± 0.028	0.241 ± 0.051
SMART (2026)	0.317 ± 0.058	0.195 ± 0.039	0.266 ± 0.040	0.406 ± 0.024	0.309 ± 0.029
SpaMI (2025)	0.201 ± 0.007	0.144 ± 0.014	0.327 ± 0.025	0.469 ± 0.028	0.253 ± 0.024
SpaMosaic (2026)^a^	0.138 ± 0.004	0.059 ± 0.034	0.254 ± 0.016	0.351 ± 0.015	0.230 ± 0.075
CoMo (2026)	0.280 ± 0.051	0.167 ± 0.016	0.243 ± 0.018	0.355 ± 0.059	0.156 ± 0.068
ARISE (2026)^b^	0.357 ± 0.077	0.215 ± 0.024	0.217 ± 0.025	0.239 ± 0.038	0.395 ± 0.102
SpatialCOC (2026)^c^	0.312 ± 0.004	0.165 ± 0.047	0.268 ± 0.024	0.478 ± 0.003	—
SpaMFG (2026)^d^	0.324	0.044	—	—	0.124
Established single-omics spatial methods (concatenated features)					
GraphST	0.320 ± 0.017	0.179 ± 0.003	0.340 ± 0.012	0.533 ± 0.008	0.289 ± 0.009
SpaGCN	0.298 ± 0.034	0.118 ± 0.029	0.335 ± 0.018	0.351 ± 0.057	0.136 ± 0.022
STAGATE	0.274 ± 0.036	0.116 ± 0.009	0.334 ± 0.005	0.438 ± 0.022	0.173 ± 0.014
Model-free and untrained references (not competing methods)					
Naive concatenation	0.460	0.229	0.455	0.643	0.325
Variance-scaled concatenation	0.270	0.138	0.307	0.604	0.339
MultiSpaceNet, untrained	0.324	0.173	0.371	0.406	0.278

Each method retains its published preprocessing and training path; comparability begins at the returned embedding, which is scored by one unified pipeline (L2 → PCA dimension capped at 20 → mclust EEE, seed 2020; full specification in Methods, “Evaluation pipeline”). MultiSpaceNet uses the fully *a-priori* training protocol (fixed 1000 epochs, $w_{p}=3$). Values are mean ± SD (MultiSpaceNet, SpatialGlue and the established spatial methods over 10 seeds; the recent stochastic multi-omics methods over 5 seeds); SpaMFG uses its released fixed seeds and is reported as one deterministic run. Bold indicates the best result per dataset among the published methods. The last three rows are references rather than competing methods, scored through the identical pipeline: a concatenation of the two feature matrices with no model at all, the same concatenation with each modality block rescaled to unit total variance, and the identical architecture with the training loop skipped (10 seeds); they calibrate what the shared pipeline itself contributes (see text), and the naive concatenation is higher than MultiSpaceNet on Spleen Rep1. The untrained row prints the untrained arm of the ten-seed paired run of Table S18, whereas the MultiSpaceNet row is an independent training run, so a trained-minus-untrained difference formed within this table (+0.162) mixes two runs and understates the matched contrast (+0.179), whose arms share seeds and initial weights; the two trained runs differ mainly on the bimodal Lymph Node (see text).

a SpaMosaic is a cross-sample/mosaic batch integrator; on single-slide domain identification its design target differs, and it is used in its intended role as the primary comparator in the integration analysis (Results, “Cross-sample integration”).

b ARISE uses its official preprocessing, graph builder, model, loss and fixed 350-epoch schedule, but the final-epoch embedding is scored because its released helper’s best-epoch rule consults ground-truth ARI.

c SpatialCOC is evaluated on its four officially supported shared mouse datasets; its released preprocessing does not accept the 10× Visium CITE-seq lymph-node input, so that cell is left blank.

d For SpaMFG, mclust EEE returned no fitted model on either thymus embedding; those cells are left blank, while its released Leiden scores (0.186 and 0.304) are reported in Table S14 and are not substituted here. Single-omics methods (GraphST, SpaGCN, STAGATE) receive the concatenated RNA+ protein features.

The last three rows of Table 2 calibrate these absolute numbers. A concatenation of the two feature matrices with no model at all reaches mean ARI 0.422 through the identical pipeline, and the untrained network 0.310: a substantial part of the absolute ARI attainable on these benchmarks is carried by the shared preprocessing and by mclust at the ground-truth *K* rather than by any model. The margin over the published methods, not the absolute score, is therefore the quantity we interpret, and the controlled counterpart is the trained-versus-untrained contrast under matched conditions (Table S18).

Qualitatively, MultiSpaceNet reproduces the annotated tissue architecture across platforms. On the Human Lymph Node, comparing all twelve maps side by side (Fig. 2) shows that lower ARI corresponds to either smooth but misplaced boundaries or fragmented compartments rather than one uniform failure mode: SMART and GraphST remain spatially smooth but misassign the germinal-centre core, while several of the lower-scoring methods fragment or merge compartments. The same holds on the other platforms: on the SPOTS spleen the model delineates white pulp, red pulp and marginal zone, and on the Stereo-CITE-seq thymus it resolves cortex, medulla and specialized regions (full per-dataset ground-truth-versus-prediction maps and UMAP embeddings in Supplementary Figs. S1 and S2).

**Figure 2:**
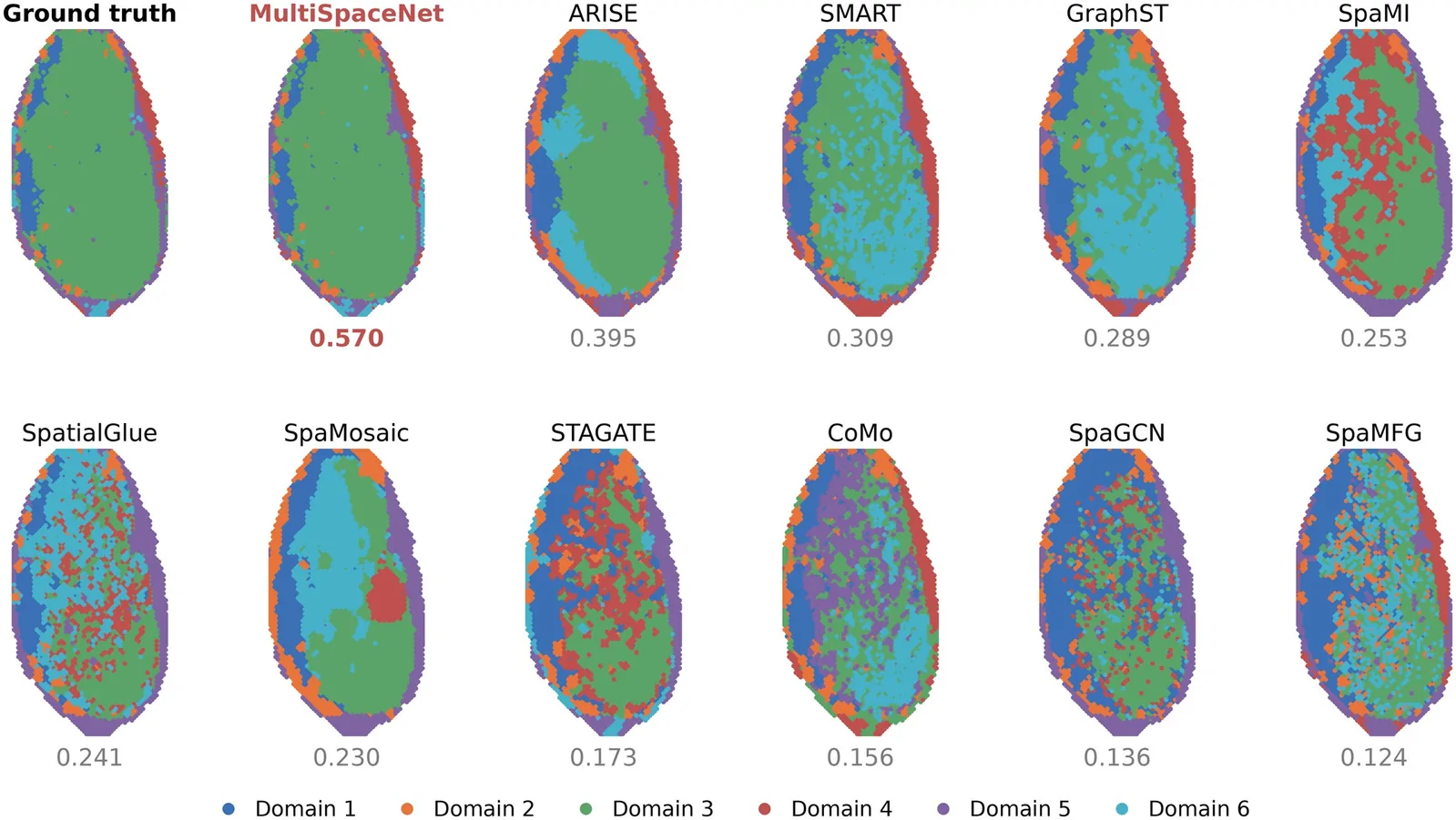
Spatial domain maps on the Human Lymph Node, the highest-variance benchmark: ground truth, MultiSpaceNet and the ten baselines evaluated on this dataset (the nine common-protocol baselines plus SpaMFG), ordered by ARI (multi-seed mean beneath each map; predicted labels are matched to ground truth by Hungarian assignment, so the shared domain key below applies to every panel). MultiSpaceNet has a bimodal seed distribution here (Methods, “Evaluation pipeline”), so its displayed map is from the upper mode while the number beneath it is the 10-seed mean; SpaMFG is a deterministic fixed-seed result and the other numbers are method means. The other baseline maps are single representative runs: the seed-0 run for SpatialGlue, SMART, SpaMI, SpaMosaic, GraphST, SpaGCN and STAGATE, and the run closest to the method mean for CoMo and ARISE. MultiSpaceNet recovers the germinal-centre/B-follicle core and the surrounding T and medullary zones; the baselines degrade in two distinct ways, described in the text. Quantitative comparison across all five datasets: Table 2; SpatialCOC is reported there on the four shared mouse datasets supported by its official preprocessing and has no lymph-node map. Per-dataset maps, UMAP embeddings and per-seed distributions: Supplementary Figs. S1–S3.

Performance stability is dataset-dependent. The mouse datasets show comparatively narrow seed-to-seed spread (box-plus-strip distributions in Fig. S3): Spleen Rep1, Spleen Rep2 and Thymus Rep1 are tight, and Thymus Rep2 is tight in its dominant mode aside from two low-outlier seeds, indicating largely reproducible results across random initializations. The Human Lymph Node, in contrast, is the highest-variance benchmark (σ=0.214): its ARI follows a bimodal seed distribution (median 0.463, interquartile range [IQR] [0.433,0.772]). We therefore report its median and IQR rather than emphasize the percentage gain; the median still exceeds the best baseline and even the lower mode (ARI ≈0.43) is competitive with it, and we discuss this optimization-stability behaviour explicitly in the Limitations.

To separate what the training contributes from what the architecture provides as a fixed feature map, we also scored the identical network with the training loop skipped (Fig. 3a; Table S18). Mean ARI rises from 0.310 untrained to 0.489 trained, improving on all five datasets (10 seeds per arm, the two arms sharing their seeds). The trained arm is one of four independent ten-seed runs of the shipped configuration in this paper, alongside the benchmark run (0.472, Table 2), the ablation baseline (0.468, Table S6) and the no-graph control run (0.464, Table S21); the spread among the four is dominated by the bimodal Lymph Node (0.511–0.615 across the runs, while no mouse dataset varies by more than 0.033), and every trained-versus-variant contrast in this paper is computed within one run, never across runs. The same contrast on cross-modal structure preservation improves on all five as well (Fig. 3b), and across replicate sections the full model is ahead of a matched model with message passing removed in all three scenarios (Fig. 3c and d; Results, “Cross-sample integration”). Together these are the paper’s central evidence: each is a comparison against a modified version of the same model, so what they measure is the model’s own contribution rather than a difference between implementations.

**Figure 3:**
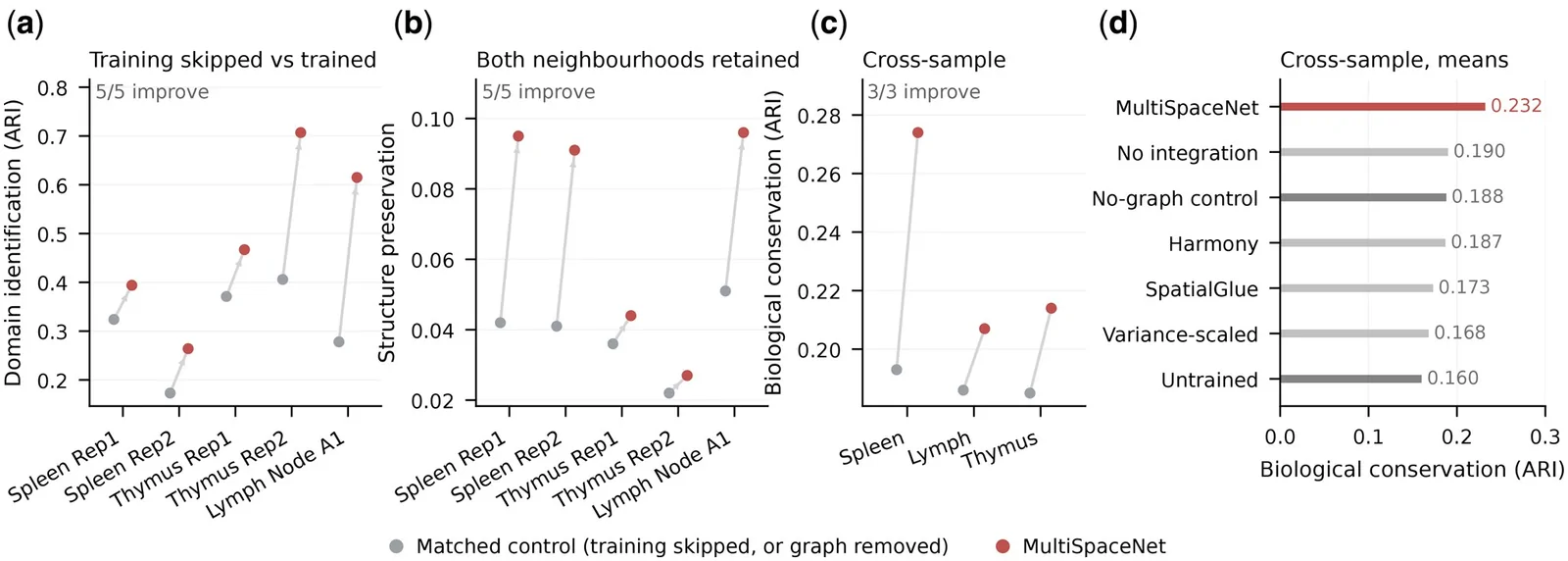
The learned contribution, isolated by matched counterfactuals. Panels (a–c) compare the model against modified versions of itself, so what is measured is the model’s own contribution; panel d then places the full model among the published comparators and model-free references on the same axis. (a) Spatial-domain identification (ARI) with the training loop skipped versus trained, on all five datasets; one line per dataset, 10 seeds per arm sharing their seeds (Table S18). (b) Cross-modal structure-preservation balance $\text{min}(J_{\text{RNA}},J_{\text{prot}})$ for the same contrast and the same five datasets, showing that training makes the embedding retain both modalities’ neighbourhoods rather than collapsing onto the transcriptome. (c) Cross-sample biological conservation (ARI) with message passing removed versus the full model, over the three replicate-section scenarios (Table S20). (d) Where the full model sits on that axis against the published comparators and the model-free references, as dataset means; the no-graph control shares this model’s objective, encoders and attention fusion and differs only in that the typed graph and the SAGEConv layer are removed. Dark grey marks this model’s own matched controls (no-graph, untrained); light grey the published comparators and model-free references. Bars start at zero. Source values are transcribed from the cited Supplementary Tables.

One question these controls leave open is whether the gain they isolate is particular to this formulation or would be found in other models of this kind. We therefore ran the same control on SpatialGlue and SMART and report it as a descriptive implementation diagnostic rather than novelty evidence: each embedding taken from the freshly initialized network under that method’s own code, with the training loop skipped and nothing else changed, scored against its own trained embedding through the identical pipeline, ten seeds per arm (Table S24). Training raises MultiSpaceNet’s mean domain ARI by 0.179, improving on all five datasets; SMART’s by 0.054, also on all five; and SpatialGlue’s by 0.013, on three of five. Because the two arms of a method share their seeds, Δ is paired and its dispersion is reported per dataset in that table.

We do not use differences among these changes as novelty evidence. The models have different objectives and parameterizations, so their trained-minus-initialized values do not measure a common “training signal”; moreover, the across-method unit is only five datasets. We therefore report directions and effect sizes without a cross-architecture significance test or ratio. As a reproduction check, both comparators’ trained means here (0.298 and 0.317) reproduce their Table 2 values. The only supported inference is within MultiSpaceNet: under its own matched initialization control, optimization materially changes the representation and its downstream score. That is a necessary training sanity check, not a claim of methodological novelty or superior use of a commensurate training signal.

### 3.3 Ablation study

We ablate architectural components and edge types on all five datasets over 10 seeds, reporting mean ± SD and a paired Wilcoxon test against the full model; the complete tables are in Supplementary Tables S6 and S7. Because an ablation is only as informative as the metric it is scored on, we additionally re-scored every variant on two further axes—leakage-free cross-modal prediction and structure-preservation balance—rather than on ARI alone (Table S19).

No single component is established as individually necessary: most effects fall within seed-to-seed variability, and only three of the twenty-five component-ablation variant×dataset comparisons reach significance (the edge-type table adds one further significant effect). The five-dataset means, printed in Supplementary Tables S6 and S7, make the point sharper than the per-dataset cells do: within the component-removal block the shipped configuration (0.468) is behind removing the cross-modal decoder (0.478); the separate network-depth block scores higher still (one layer 0.482, three layers 0.490); and on the edge axis a graph with the spatial relation deleted (0.474) is ahead of the full three-relation graph (0.468). We print these means because they state this observation in its strongest form. Removing HANConv significantly lowers ARI on Spleen Rep2 alone (0.264→0.214, *P* < .01); on Spleen Rep1 two simplifications—removing SAGEConv, or using three HANConv layers—slightly but significantly *raise* it (0.373→0.404 and 0.413), though neither helps consistently (*w/o SAGEConv* is the weakest variant on the Lymph Node). Removing the cross-modal decoder is never significant (e.g. Lymph Node *P* = .92). No HANConv depth is uniformly best, so we retain two layers as a default that requires no per-dataset architecture search.

The edge-type ablation tempers the architectural claim similarly: on point estimates a *single* relation type ties or exceeds the full three-relation graph on four of five datasets, and the full graph is clearly best only on Spleen Rep2, where dropping to a spatial-only graph significantly reduces ARI (0.264→0.233, *P* < .01)—the single significant edge-type effect. A matched test in the cross-sample integration regime likewise finds no significant advantage of the full graph over the best single relation (Table S11). We therefore present the full configuration as a *unified default*—which ranks first in Table 2—rather than as a set of individually necessary components.

### 3.4 Modality attention interpretation

The cell-level attention mechanism in MultiSpaceNet provides a descriptive attribution of the two modality branches across tissues and platforms. We extract the learned weights $\alpha_{\text{RNA}}$ and $\alpha_{\text{Protein}}$ for each cell and aggregate them by dataset and spatial domain (per-dataset modality-dominance maps in Fig. S4).

The two SPOTS mouse spleen datasets exhibit near-balanced weights with a slight protein lean. The RNA/protein means are 0.46/0.54 in both replicates. In contrast, the two Stereo-CITE-seq mouse thymus datasets show a pronounced RNA bias: 0.65/0.35 in Rep1 and 0.66/0.34 in Rep2. The human lymph node (10× Visium + CITE-seq) is likewise near-balanced (0.48/0.52). These descriptive patterns coincide with platform differences—the Stereo-CITE-seq data combine transcriptome-wide RNA with targeted 19–51-marker protein panels—but the weights alone do not establish why a branch is favoured or how much biological information it contributes.

The per-cell modality-dominance maps (Fig. S4) are spatially heterogeneous within each tissue: the thymus datasets display gradients under overall RNA dominance, while the near-balanced spleen and lymph node retain tissue-scale variation. Three controls delimit their meaning. First, replacing per-cell weights at inference with a dataset-global mean does not significantly change clustering (pooled paired Wilcoxon *P* = .60; Table S12). Second, protein-weight maps are spatially autocorrelated (Moran’s *I* in 0.30–0.59, permutation *P* = .05), but the untrained control shows that some smoothness is inherited from the inputs. Third, in a pre-specified stress test that replaces one modality within a contiguous 30% spatial patch, the weights do not reliably identify or downweight the corrupted branch: across 25 dataset–seed runs per modality, attention AUC is 0.511±0.079 for RNA corruption and 0.420±0.082 for protein corruption (Table S25). We therefore treat these maps as descriptive model attributions, not causal explanations, quality estimates, or evidence of adaptive robustness. Because the two weights sum to one, the RNA map is the exact complement of the protein map and is not reported separately.

### 3.5 Cross-modal structure preservation

A joint embedding of two modalities should retain both of their neighbourhood structures rather than collapse onto whichever modality contributes more features. We measure this directly: for each representation we take the k=30 nearest-neighbour graph of the embedding together with those of the RNA-only and protein-only feature spaces, and report the Jaccard overlaps $J_{\text{RNA}}$ and $J_{\text{prot}}$ and their minimum—the balance, which is high only when *both* are preserved. All methods are scored with identical metric code (Table S16).

Naive concatenation is dominated by the transcriptome ($J_{\text{RNA}}=0.71$ against $J_{\text{prot}}=0.046$). The cause is scale, not dimensionality: both modalities enter as equal numbers of principal components, but the RNA block carries 5.6–47.7× the total variance. MultiSpaceNet trades RNA overlap for higher protein overlap (0.415/0.071 on its five-dataset mean). Comparator cell sets are identical to ours on Spleen Rep1, Spleen Rep2 and the Lymph Node, but not on the two thymus sections; we therefore compare methods on that common three-dataset subset only. There, the seed-0 balance is 0.092 for MultiSpaceNet, 0.063 for SMART, 0.048 for SpatialGlue, 0.036 for SpaMI and 0.037 for SpaMosaic (Table S16). The two overlaps and unequal coverage are printed explicitly (Supplementary Tables S23 and S22); no thymus ranking is claimed. Because the minimum can bind on different modalities for different methods, balance is a diagnostic of joint neighbourhood retention rather than a general representation-quality score.

The metric also has a ceiling that no model is needed to reach. Rescaling each modality block to unit total variance before concatenating—two lines of arithmetic, no model and no training—attains a five-dataset mean balance of 0.173, above every learned representation here. Yet that representation has mean spatial-domain ARI 0.332, against 0.422 for naive concatenation and 0.472 for MultiSpaceNet. Balance can therefore be maximized without learning and is not a utility ranking. Its defensible use here is narrower: at the fixed $w_{p}=3$ setting, training the same network increases retention of the limiting modality on every dataset.

We scored the identical network with training skipped, using the same initial weights, preprocessing and evaluation (Table S18). Over ten seeds per arm, training raises balance from 0.038 to 0.071 and domain ARI from 0.310 to 0.489, with both quantities increasing on every dataset. The dataset-level balance changes are 0.042→0.095, 0.041→0.091, 0.036→0.044, 0.022→0.027 and 0.051→0.096. Attention Moran’s *I* rises from 0.275 to 0.426 on the five-dataset mean and on every dataset, but the untrained network already reaches 0.275, so spatial smoothness is partly inherited from the inputs. This control isolates a positive optimization contribution relative to the initialized network; it does not identify heterogeneous relation typing as the cause, establish component necessity, or provide evidence of architectural novelty.

Two further controls bound the graph claim. A matched no-graph autoencoder reaches mean ARI 0.471 against 0.464 for the full model within sections, so that comparison is inconclusive. In the joint replicate-section setting, however, the full graph model has higher biological-conservation ARI than the matched no-graph model in all three scenarios (0.232 versus 0.188 on the scenario mean; Fig. 3c and d). We therefore locate the graph-based stage’s positive evidence on cross-section embedding, not on within-section clustering, and do not attribute it specifically to three-relation typing. Finally, balance depends monotonically on the protein-loss weight (0.026, 0.044 and 0.071 at $w_{p}=0,1,3$; Table S21), reinforcing its role as a diagnostic rather than an objective method ranking.

### 3.6 Cross-modal protein expression prediction

Because MultiSpaceNet keeps one encoder per modality and fuses only at the cell-level attention step, its pre-fusion RNA embedding $h_{\text{RNA}}\in R^{N\times 128}$ remains a function of RNA alone even though it is trained under the joint objective. That makes a practical read-out available: predicting protein abundance where no protein was measured. We report it as such, and we are careful about what it does and does not demonstrate.

The task is not well posed for every integration method. SMART, SpaMI and SpaMosaic fuse the modalities at their input, so their joint embedding is itself a function of the measured protein and predicting that protein from it recovers the input rather than imputing it. Run anyway under the identical protocol, SMART reaches 99.4% (0.8235/0.8287) of what a Ridge achieves when handed the protein features directly, against 0.531 for a Ridge with no protein access (Table S17). The comparison below is therefore restricted to modality-separable representations—MultiSpaceNet’s $h_{\text{RNA}}$ and SpatialGlue’s RNA_latent ($R^{N\times 64}$)—together with a Ridge and a non-linear MLP on raw RNA and the two purpose-built generative CITE-seq imputers totalVI and sciPENN. For each representation we train a Ridge regression model with five-fold cross-validation to predict log-normalized protein expression (Table 3; extended baselines in Table S15).

**Table 3:** Cross-modal protein prediction under a *leakage-free* inductive five-fold protocol (per fold the encoder is retrained on training cells only).

Dataset	MultiSpaceNet	SpatialGlue	raw-RNA	Win (signal)
Spleen Rep1	**0.604**	0.542	0.562	19/21
Spleen Rep2	**0.651**	0.547	0.582	20/21
Thymus Rep1	**0.574**	0.549	0.550	15/18
Thymus Rep2	**0.599**	0.500	0.526	19/19
Lymph Node A1	0.438	0.419	0.437	25/31

Mean Pearson *r* over signal-bearing proteins (max r>0.1); a raw-RNA Ridge baseline (inherently leakage-free) is included as a no-protein-access reference. “Win” = signal-bearing proteins where MultiSpaceNet exceeds SpatialGlue. Bold: highest mean *r* of the three sources (near-ties with the raw-RNA baseline, as on the lymph node, are left unbolded and noted in the text).

As shown in Table 3 and Fig. S8, under the leakage-free protocol MultiSpaceNet’s RNA embedding wins on 98/110 (89.1%) signal-bearing protein targets against SpatialGlue’s RNA_latent, and exceeds a raw-RNA Ridge baseline on four of five datasets (e.g. Spleen Rep2, 0.651 versus 0.582; essentially tied on the Human Lymph Node, 0.438 versus 0.437). The original leaky protocol differs from the leakage-free result by ≤0.016 on every dataset, so removing the leakage does not change the numbers.

That margin over raw RNA should be read with the structure of the probe in mind. $h_{\text{RNA}}$ is a 128-dimensional non-linear expansion of 20 RNA principal components, so a Ridge fitted on it is more expressive than a Ridge on the components themselves. We therefore read the comparison as accuracy at imputing protein rather than as evidence about the quality of the learned representation. To check that the cross-modal signal is not specific to a linear probe or to the choice of SpatialGlue as the sole comparator, we added, under the identical folds and target, a non-linear MLP on raw RNA and the two purpose-built generative CITE-seq protein-imputation methods totalVI and sciPENN (Table S15). MultiSpaceNet’s mean signal-bearing *r* (0.573) exceeds the MLP (0.504), totalVI (0.225) and sciPENN (0.323) on all five datasets. We read this carefully: totalVI and sciPENN output a compositional, per-cell-normalized protein, so scoring them against the measured log-count target reflects accuracy at *recovering measured abundance* (the practical imputation use-case) rather than a like-for-like generative benchmark. That the direct per-protein predictors (MultiSpaceNet, Ridge) lead is consistent with the established advantage of supervised prediction over amortized generative imputation (Lakkis *et al.* 2022).

Figure 4d (larger renderings in Fig. S8) compares measured and predicted spatial expression for three biologically representative markers on the Human Lymph Node dataset. T-cell marker CD3E (leakage-free Pearson r=0.733) is accurately localized to T zones, B cell marker MS4A1/CD20 (r=0.468) clusters in follicular regions, and mesenchymal marker VIM (r=0.683) delineates stromal architecture (further markers such as macrophage CD68, r=0.628, in Table S5). The predicted maps recover the spatial organization of each marker, which is what matters for the use case: where protein was not measured, the RNA branch supplies a spatially faithful surrogate. As noted above, $h_{\text{RNA}}$ is a non-linear expansion of the RNA principal components, so we present these maps for their practical value in imputing protein where none was measured rather than as a ranking of representations. Predicted-versus-actual scatter plots for representative markers are shown in Fig. S5. Complete per-protein results are listed in Supplementary Tables S3–S5 for four of the five datasets; Mouse Thymus Rep1 is summarized in the accompanying Supplementary note (mean r=0.574, 15/18 signal-bearing wins).

**Figure 4:**
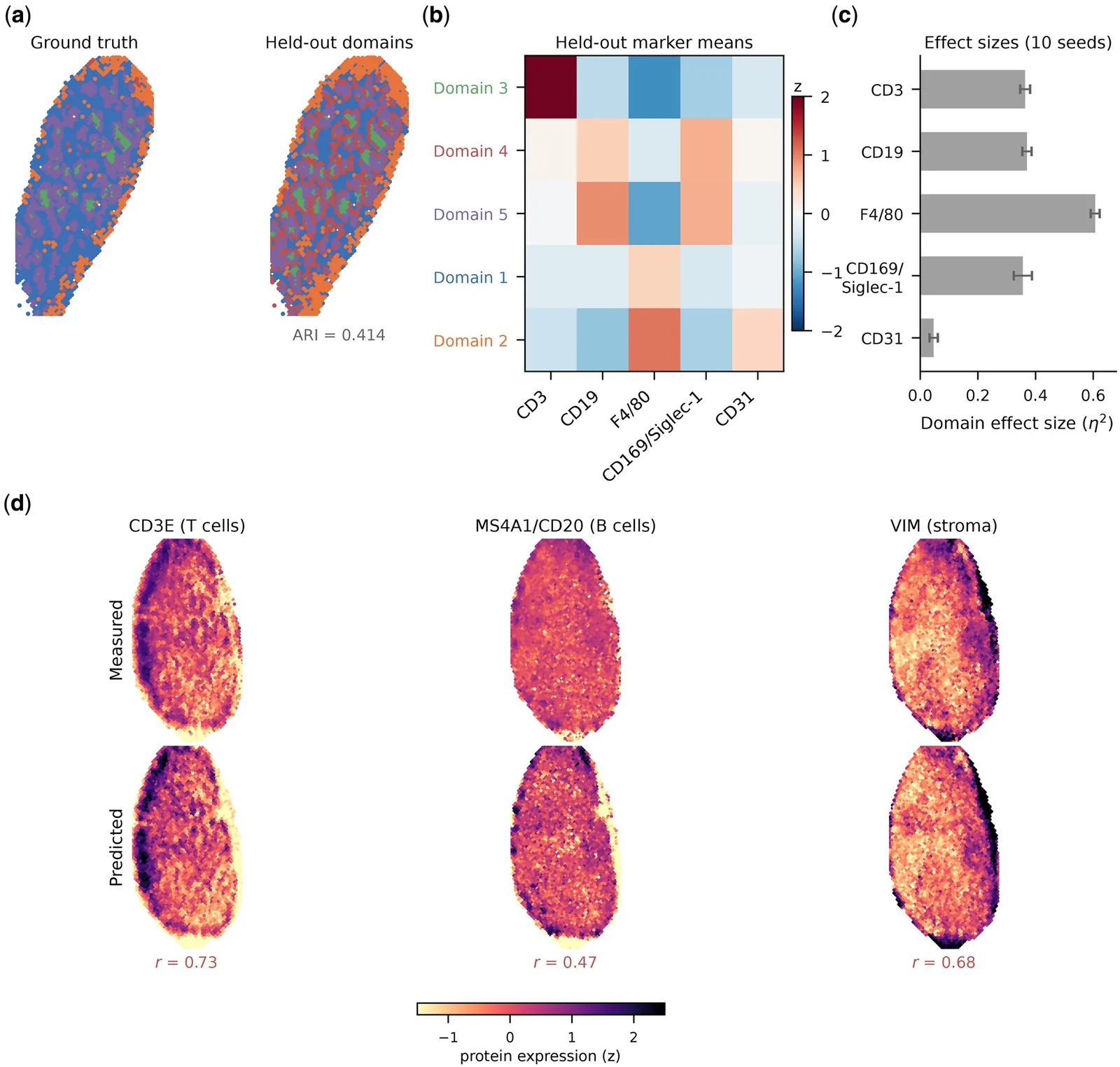
Leakage-free protein read-outs. Top row, Mouse Spleen Rep1: held-out protein-marker validation. The five displayed proteins were excluded before protein preprocessing and from every model input, graph and loss. (a) Ground-truth annotation beside the spatial domains of the representative seed 0 held-out-input model (ARI =0.414); predicted labels are matched to ground truth so the two maps share colors. (b) Mean log-transformed held-out marker expression in each predicted domain, z-scored per marker across cells; domain rows are hierarchically ordered for display only and their labels are colored as in (a). (c) Domain effect size $\eta^{2}$ for each marker, mean±SD over ten independently trained seeds. CD3 (T cells), CD19 (B cells), F4/80 (red-pulp macrophages) and CD169/Siglec-1 (marginal-zone macrophages) are strongly partitioned; the CD31 (endothelium) association is weak and is reported without a compartment claim. No domain was assigned to a marker by optimization. Bottom row, Human Lymph Node: (d) measured versus leakage-free out-of-fold predicted spatial expression for three representative markers, from the same inductive five-fold protocol and folds as Table 3 (Pearson *r* against the measured protein beneath each prediction; larger renderings in Fig. S8). The two rows are different datasets and protocols: (a–c) validate domains against proteins the model never saw, (d) is the imputation read-out of the trained model.

### 3.7 Biological validation of the predicted spatial domains

To avoid reusing a model input as biological evidence, we repeated the Mouse Spleen Rep1 analysis with five validation proteins fixed in advance—CD3 (T zone), CD19 (B follicle), F4/80 (red-pulp macrophages), CD169/Siglec-1 (marginal zone) and CD31 (vascular/stromal). These five columns were removed *before* protein normalization and PCA and were absent from the protein-similarity graph, both reconstruction targets and the RNA-to-protein loss. They were read only after the embedding had been clustered. No marker-to-domain assignment or Hungarian matching was used.

The held-out-input model retains spatial-domain structure over ten training seeds (ARI 0.414±0.030). Four markers show substantial partitioning by the predicted domains: mean $\eta^{2}=0.364$ for CD3, 0.370 for CD19, 0.607 for F4/80 and 0.356 for CD169/Siglec-1; CD31 is weaker (0.046) and we do not interpret it as a recovered compartment (Fig. 4; Table S8). The representative heat map shows which domains carry each held-out signal without forcing a one-to-one anatomical assignment. Thus, the result is an input-independent plausibility check that the learned partition aligns with unobserved immune and macrophage protein patterns, not evidence of a new biological discovery or of superiority over another method.

### 3.8 Cross-sample integration: what MultiSpaceNet does and does not do

Spatial-domain ARI measures clustering quality on a single section; it does not, by itself, test *integration*. To characterize MultiSpaceNet as an integration method we ran a dedicated cross-sample analysis on three within-platform scenarios whose replicates share a feature space—Mouse Spleen Rep1 + Rep2 (21 shared proteins), Human Lymph Node A1 + D1 (30 shared proteins), and Mouse Thymus Rep2 + Rep3 + Rep4 (19 shared proteins); the Thymus Rep1 antibody panel is disjoint from the others and is therefore excluded from multi-omics cross-sample integration. Thymus Rep3/Rep4 and Lymph D1 are additional replicate sections from the same source studies (Stereo-CITE-seq mouse thymus and 10× Visium CITE-seq human lymph node, respectively); they are used only for this cross-sample analysis and are not part of the single-section benchmark of Table 1. Each method embeds the replicates jointly and is scored with standard scIB (single-cell integration benchmarking) metrics under one protocol: batch removal (iLISI, integration local inverse Simpson’s index; kBET, *k*-nearest-neighbour batch-effect test; ASW${\;}_{\text{batch}}$, batch average silhouette width; and a batch-classifier probe) and biological conservation (ARI; NMI, normalized mutual information; cLISI, cell-type local inverse Simpson’s index; ASW${\;}_{\text{label}}$; graph connectivity). We compare against no integration (joint PCA), Harmony, and SpaMosaic—a dedicated spatial mosaic/batch integrator (Table S9).

The results reveal a clear batch-removal versus biology-preservation trade-off (Fig. 5; full scIB metrics in Table S9). The dedicated integrators remove batch most strongly (Harmony and SpaMosaic reach batch scores of 0.64–0.85), whereas MultiSpaceNet performs essentially no batch removal (its batch score is at or below no integration on two of three scenarios). Of the four methods, MultiSpaceNet has the *highest* bio-conservation in every scenario (bio 0.596/0.581/0.544; bio-ARI 0.273/0.207/0.214), and SpaMosaic—despite being purpose-built for spatial mosaic integration—the lowest. Because the standard scIB composite weights batch removal at 0.4, the two dedicated integrators lead on that composite (Table S9); we report this openly rather than headline only the sub-metric we win. The bio advantage over no integration is small but consistent on the mean (positive in every scenario, paired *t*-test *P* = .048, and on 14 of 15 individual seed runs on the scIB biological-conservation composite; the 12/15 and 13/15 figures quoted below are for the bio-ARI sub-metric). We therefore make a deliberately bounded claim: MultiSpaceNet best preserves biological (spatial-domain) structure when jointly embedding replicate sections, but it is *not* a batch-correction method and does not lead on the batch-weighted composite. Because the domain-identification and structure-preservation axes are reported alongside model-free and untrained controls, we apply the same standard here, and this is the axis on which those controls are cleared by the widest margin (Table S20). The comparator set was extended at review in three ways: SpatialGlue, run on the same joint sections with its own preprocessing; the matched no-graph autoencoder, which shares this model’s objective, encoders and attention fusion and removes only the typed graph and the SAGEConv layer; and two model-free references. MultiSpaceNet has the higher biological conservation in all three scenarios against all six. All values in this comparison come from one re-evaluation run (Table S20), which reproduces Table S9’s MultiSpaceNet values to within rounding (Spleen 0.274 against 0.273, identical elsewhere), so every contrast here is within-run: mean bio-ARI 0.232, against the no-graph autoencoder 0.188, SpatialGlue 0.173, no integration 0.190, Harmony 0.187, the variance-scaled concatenation 0.168 and the untrained network 0.160. The no-graph comparison is the one we would draw attention to, because it is the control, i.e. *indistinguishable* from the full model on the within-slide axes (Table S21): here it is not, which is why we locate the contribution on this axis rather than on within-slide clustering. The unit that generalizes here is the scenario, not the seed, and the direction is consistent across all three for every comparator. We also give seed-level paired tests, but as a measure of optimization stability rather than of replication: pooling the fifteen runs (three scenarios × five seeds) gives win rates of 13/15 against the no-graph autoencoder (*P* = .0034), 13/15 against SpatialGlue (*P* = .0026), 12/15 against no integration (*P* = .0020), 12/15 against Harmony (*P* = .0012), 12/15 against the variance-scaled concatenation (*P* = .0034) and 15/15 against the untrained network (*P* = .0001). These are not fifteen independent biological replicates and we do not read them as such. SMART and SpaMI are single-section methods whose released code we could not instantiate for the joint-section setting, and we say so rather than imply wider coverage. The last of these is the one that carries the attribution: the untrained control shares the architecture, the preprocessing and the scoring code, so what separates the two arms is the self-supervised training itself. Cross-platform integration (e.g. spleen versus thymus) is excluded as a biology/platform confound rather than a fair batch test.

**Figure 5:**
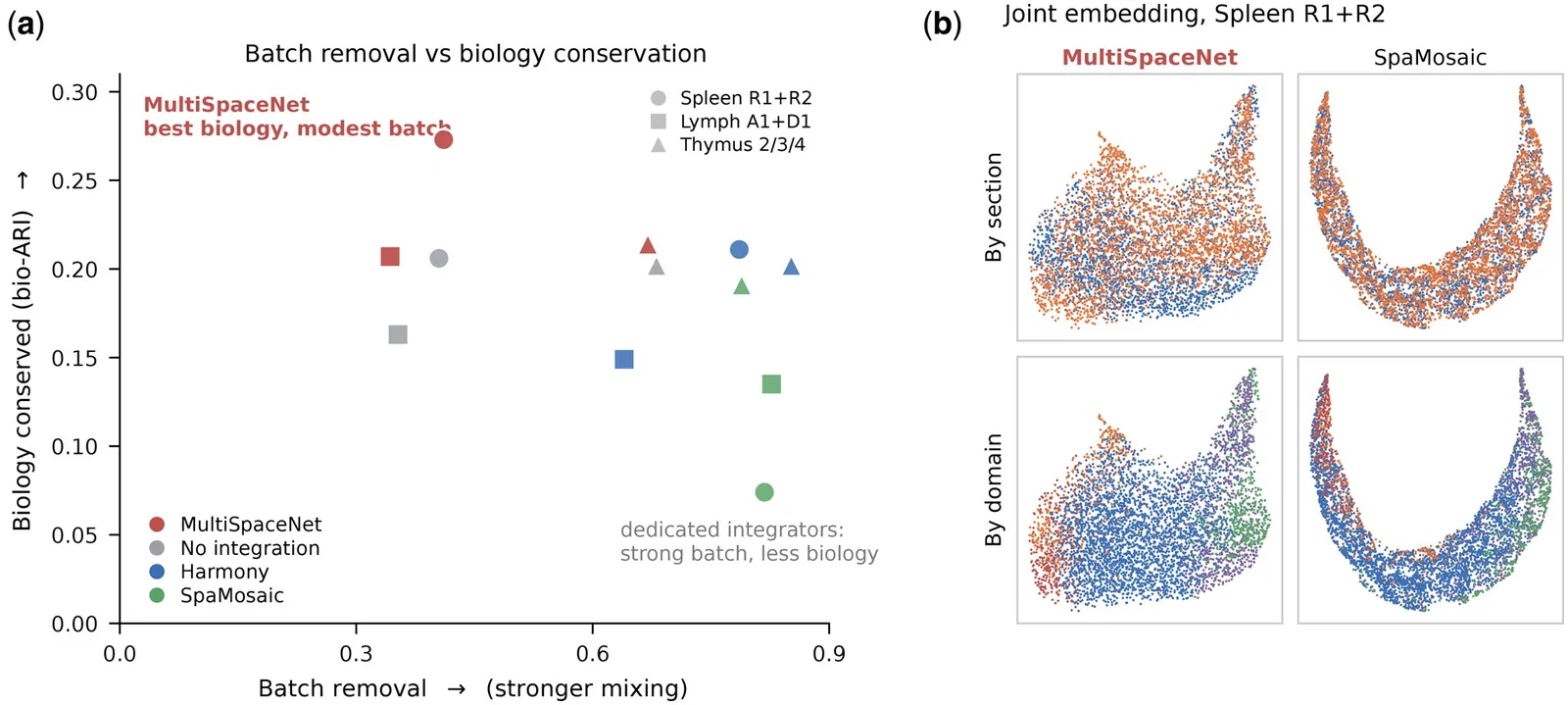
Cross-sample integration is a batch-removal versus biology-preservation trade-off. (a) Batch removal (*x*) versus biological conservation (bio-ARI, *y*) for every method and scenario (marker shape encodes the scenario). The batch-removal axis is the scIB batch composite of Table S9: the mean of the iLISI, kBET, batch-ASW and batch-classifier scores. MultiSpaceNet (red) attains the highest biological conservation but performs little batch removal, whereas the dedicated integrators Harmony and SpaMosaic reach stronger batch removal at lower biological conservation—a clear trade-off (on the batch-weighted scIB composite the dedicated integrators lead; Table S9). scIB metrics, mean over 5 seeds; numbers as in Table S9. (b) Joint-embedding UMAPs for the Spleen R1+R2 scenario, MultiSpaceNet versus SpaMosaic, coloured by replicate section (top) and by ground-truth spatial domain (bottom). MultiSpaceNet keeps the domain structure coherent while mixing the sections only weakly—the same trade-off as (a) read qualitatively. The full three-scenario, four-method grid with color keys is Fig. S7.

We additionally probe modality contribution by dropping a modality at inference (5 seeds; Table S10). Dropping either modality never collapses the embedding (graceful degradation), but the stronger statement is this: for spatial-domain identification the second modality does *not* help on most datasets. RNA alone matches or exceeds the fused model on three of five datasets—significantly so on Spleen Rep1 (0.493 versus 0.368, +0.125, positive on 5/5 seeds, paired *t P* = .001)—and protein adds value only on Thymus Rep2 (0.728 versus RNA-only 0.670) and the Human Lymph Node (0.542 versus 0.478), neither reaching significance. We therefore do *not* claim that fusing the two modalities improves clustering; protein’s supported value in this study is the balanced joint representation (Results, “Cross-modal structure preservation”), while the attention map is retained only as a descriptive attribution (Results, “Modality attention interpretation”). (These figures are from the separate missing-modality probe and differ slightly from the main benchmark of Table 2, which uses independent seeds.)

## 4 Conclusion

In this work we presented MultiSpaceNet, a graph-based framework for joint representation learning from paired spatial transcriptome–proteome sections. A section is represented as a single cell graph carrying spatial proximity, RNA similarity and protein similarity as distinct relations; a cell-level attention module fuses the two modalities into one embedding, which is refined along spatial edges and trained by self-supervised reconstruction.

Under a single unified embedding-to-clustering protocol and a training budget fixed in advance, MultiSpaceNet ranks first in spatial domain identification on all five datasets against nine full-coverage published baselines, including CoMo and the topology-focused ARISE method, and also leads SpatialCOC on the four shared mouse datasets supported by its official preprocessing. ARISE is evaluated with its official 350-epoch schedule but a label-free final-epoch read-out; the July 2026 SpaMFG release is reported on all five under its native backend and on the three datasets where the common evaluator returns a fit. MultiSpaceNet is ahead on each available comparison. Its ARI improvements over SpatialGlue are +15% to +92% under the matched protocol (Wilcoxon *P* = .014).

Matched controls define what the architecture does and does not contribute. With ten seeds per arm, scoring the same network before and after training raises mean domain-identification ARI from 0.310 to 0.489 and structure-preservation balance from 0.038 to 0.071, with both increasing on all five datasets. This is a training sanity check, not evidence that the components are novel. Within individual sections, a matched model with message passing removed has a similar five-dataset mean ARI (0.471 versus 0.464 in the independent control run). Across jointly embedded replicate sections, in contrast, the full graph model preserves biological structure better than the matched no-graph model in all three scenarios (mean bio-ARI 0.232 versus 0.188). The graph-based stage therefore has a conditional advantage for joint replicate-section embedding, not a demonstrated universal advantage for within-section clustering.

We do not claim a new graph operator or that relation typing is necessary: single-relation variants tie or exceed the full three-relation graph on most within-section datasets and in all three cross-section scenarios. The multi-relational graph is the problem formulation, not an isolated source of gain. The per-cell modality weights are likewise a descriptive read-out: replacing them with one global weight does not significantly change clustering, and under local modality corruption they do not reliably identify or downweight the damaged branch. The modality-separable RNA branch supports leakage-free protein imputation, which is reported as a practical within-section route rather than evidence of representation quality. Finally, an input-independent spleen experiment excludes five validation proteins from every model input, graph and loss; the resulting domains remain stable across ten seeds and partition four of the five held-out immune/macrophage markers strongly.

MultiSpaceNet is therefore best viewed as a joint-representation framework for paired spatial RNA–protein sections. Its supported uses are spatial-domain identification, a modality-attribution map, within-section RNA-to-protein prediction and biology-preserving joint embedding of replicate sections. It is not a general batch-correction tool: dedicated integrators lead on batch removal and on the batch-weighted scIB composite. Nor does the second modality universally improve clustering—RNA alone matches or exceeds the fused model on three of five datasets. Cross-modal structure balance is reported only on matched cell sets and as a diagnostic, because it depends on the protein-loss weight and a variance-scaled concatenation reaches a higher value without learning.

Several limitations should be noted. First, the framework targets paired transcriptomics and proteomics; extending it to additional modalities such as chromatin accessibility or metabolomics would require changes to graph construction and the encoders. Second, the bimodal seed behaviour on the Human Lymph Node dataset indicates that training stability on particularly challenging benchmarks is an open issue; reporting median/IQR and exploring consensus clustering or curriculum learning are natural next steps. Third, we do not claim a new graph operator or that each architectural component is necessary—ablations place most component effects within seed variability—so the contribution is a unified formulation, a rigorous benchmark, and the controlled attribution of the representation’s quality to its training, rather than an architectural advance. Fourth, the cross-modal read-out is a within-section imputation route and should not be read as evidence about the learned representation; the model is trained per section and does not transfer across sections, which limits imputation to panels partially measured on the same slide. Future work will extend MultiSpaceNet to more than two modalities, improve optimization stability, and apply it to larger spatial atlases and disease tissues such as tumour microenvironments.

## Supplementary Material

btag676_Supplementary_Data

## Data Availability

The datasets analysed in this study are publicly available. The mouse spleen SPOTS dataset is available from the NCBI Gene Expression Omnibus (GEO) under accession number GSE198353. The human lymph node 10× Visium CITE-seq dataset is available under accession number GSE263617. The mouse thymus Stereo-CITE-seq dataset was obtained from the original publication (Liao *et al.* 2023). Source code is available at https://github.com/yongzhuangliulab/MultiSpaceNet, with the version used for the reported results archived at Zenodo (DOI 10.5281/zenodo.22667390; concept DOI 10.5281/zenodo.21488824). The scripts that regenerate the reported tables and figures, the pinned baseline versions and the corresponding machine-readable result summaries are included in the repository (directory resubmission/, tag v1.2-bioinformatics-resubmission) and in that archive.
