# Supplementary material for: MultiSpaceNet: graph-based joint representation learning for paired spatial transcriptome–proteome data
Source: Bioinformatics. 2026 Sep 25;42(9):btag676. doi: 10.1093/bioinformatics/btag676 (PMC13622343; doi:10.1093/bioinformatics/btag676)
Supplement: Supplementary file 1 [file btag676_supplementary_data.pdf]

Supplementary Material

Evaluation Metrics

Adjusted Rand Index (ARI).

The ARI [1] measures the agreement between predicted cluster assignments and ground-truth labels, adjusted for chance. It is defined as:

$$\mathrm{ARI} = \frac{\sum_{ij} \binom{n_{ij}}{2} - \left[\sum_i \binom{a_i}{2} \cdot \sum_j \binom{b_j}{2} \right] \Big/ \binom{N}{2}}{\frac{1}{2}\left[\sum_i \binom{a_i}{2} + \sum_j \binom{b_j}{2} \right] - \left[\sum_i \binom{a_i}{2} \cdot \sum_j \binom{b_j}{2} \right] \Big/ \binom{N}{2}}, \tag{S1}$$

where N is the total number of spots, n_{ij} is the number of spots in ground-truth class i assigned to cluster j, $a_i = \sum_j n_{ij}$, and $b_j = \sum_i n_{ij}$. ARI ranges from -1 (worse than random) to 1 (perfect agreement), with 0 indicating random labelling.

Moran's I.

We use Moran's I [2] to quantify the spatial autocorrelation of cluster assignments. A positive Moran's I indicates that similar labels tend to co-locate, reflecting spatially coherent clustering. The statistic is computed as:

$$I = \frac{N}{W} \cdot \frac{\sum_{i=1}^{N} \sum_{j=1}^{N} w_{ij}(x_i - \bar{x})(x_j - \bar{x})}{\sum_{i=1}^{N} (x_i - \bar{x})^2}, \tag{S2}$$

where w_{ij} is the spatial weight between units i and j, $W = \sum_{ij} w_{ij}$, x_i is the observed value, and $\bar{x}$ is the global mean. Moran's I ranges from -1 (perfect dispersion) to $+1$ (perfect clustering), with 0 indicating a random spatial pattern.

Pearson correlation (r).

For cross-modal protein expression prediction (Results, "Cross-modal protein expression prediction" of the main text), we report the Pearson correlation coefficient between ground-truth and predicted protein expression for each protein, averaged across 5-fold cross-validation splits.

Spatial domain maps and embeddings

Figures S1 and S2 give complementary spatial views behind the quantitative benchmark: selected method maps on all five datasets (Fig. S1), and UMAP embeddings of the MultiSpaceNet representations coloured by annotation and predicted cluster (Fig. S2). The broader set of methods available on the Human Lymph Node, including CoMo, SpaMFG and ARISE, is shown in main-text Fig. 2; all scores and coverage are in main Table 2.

ARI distribution across random seeds

Figure S3 shows the seed-to-seed distribution of ARI for MultiSpaceNet and SpatialGlue under the unified clustering protocol, illustrating both the reproducibility of the results on the mouse datasets and the bimodal seed behaviour on the Human Lymph Node.

Full modality-dominance maps

Figure S4 provides the per-cell modality-dominance maps for all five datasets, complementing the summary attention weights and Moran's I values reported in the main text (Results, "Modality attention interpretation").

Cross-modal protein prediction: predicted versus actual

Figure S5 shows predicted-versus-actual scatter plots for representative proteins in the Human Lymph Node under the leakage-free protocol, complementing the aggregate Pearson-r results reported in the main text.

Hyperparameter Configuration (a-priori protocol)

To avoid selecting hyperparameters on the evaluation metric, all five datasets use a single, dataset-independent configuration (Table S1): a fixed budget of 1,000 epochs (chosen by unsupervised loss convergence, not ARI), fixed dataset-independent loss weights ($w_r = 1$, $w_p = 3$, $w_c = 1$), and only the spatial neighbour count set by platform convention. Compared with per-dataset epoch and loss-weight selection, ARIs change by at most 0.05 under this fixed protocol.

Model architecture and optimisation details

This section gives the layer-level equations summarised in Methods (Methods, "Graph convolution and output").

Heterogeneous graph convolution (HANConv)

We apply $L = 2$ layers of Heterogeneous Attention Network convolution (HANConv) [3] to the fused representations on the heterogeneous graph. HANConv extends graph attention to heterogeneous graphs by learning separate attention coefficients for each edge type.

Node-level attention.

For each edge type $\phi \in \{s, r, p\}$ (spatial, RNA similarity, protein similarity) and each neighbour $j \in \mathcal{N}_i^{\phi}$ of node i, the attention coefficient α_{ij}^{ϕ} is computed via a multi-head attention mechanism with $H = 4$ heads. The per-type aggregated representation is:

$$\mathbf{h}_i^{(\ell+1),\,\phi} = \sum_{j \in \mathcal{N}_i^{\phi}} \alpha_{ij}^{\phi}\ \mathbf{W}^{\phi}\ \mathbf{h}_j^{(\ell)}. \tag{S3}$$

Semantic-level attention.

The outputs across edge types are then aggregated via learned semantic-level attention weights β_ϕ:

$$\mathbf{h}_i^{(\ell+1)} = \sum_{\phi \in \{s,r,p\}} \beta_\phi\ \mathbf{h}_i^{(\ell+1),\,\phi}, \tag{S4}$$

where β_ϕ is the layer's semantic-attention weight over edge type ϕ.

Residual connection.

After each HANConv layer we apply residual connection, layer normalisation, ELU activation, and dropout:

$$\mathbf{h}_i^{(\ell+1)} \leftarrow \mathrm{LayerNorm}\Big(\mathrm{ELU}\big(\mathrm{Dropout}(\mathbf{h}_i^{(\ell+1)}) + \mathbf{h}_i^{(\ell)}\big)\Big). \tag{S5}$$

SAGEConv spatial refinement

After the two HANConv layers, we apply an additional SAGEConv [4] layer *exclusively on the spatial subgraph* $\mathcal{E}_s$ to refine cell representations with a dedicated round of spatial smoothing:

$$\mathbf{z}_i = \mathrm{LayerNorm}\Big(\mathrm{ELU}\big(\mathrm{Dropout}(\mathrm{SAGEConv}(\mathbf{h}, \mathcal{E}_s)) + \mathbf{h}_i\big)\Big). \tag{S6}$$

This spatial-specific refinement step adds a dedicated round of smoothing on the spatial subgraph, complementing the multi-relational message passing of HANConv.

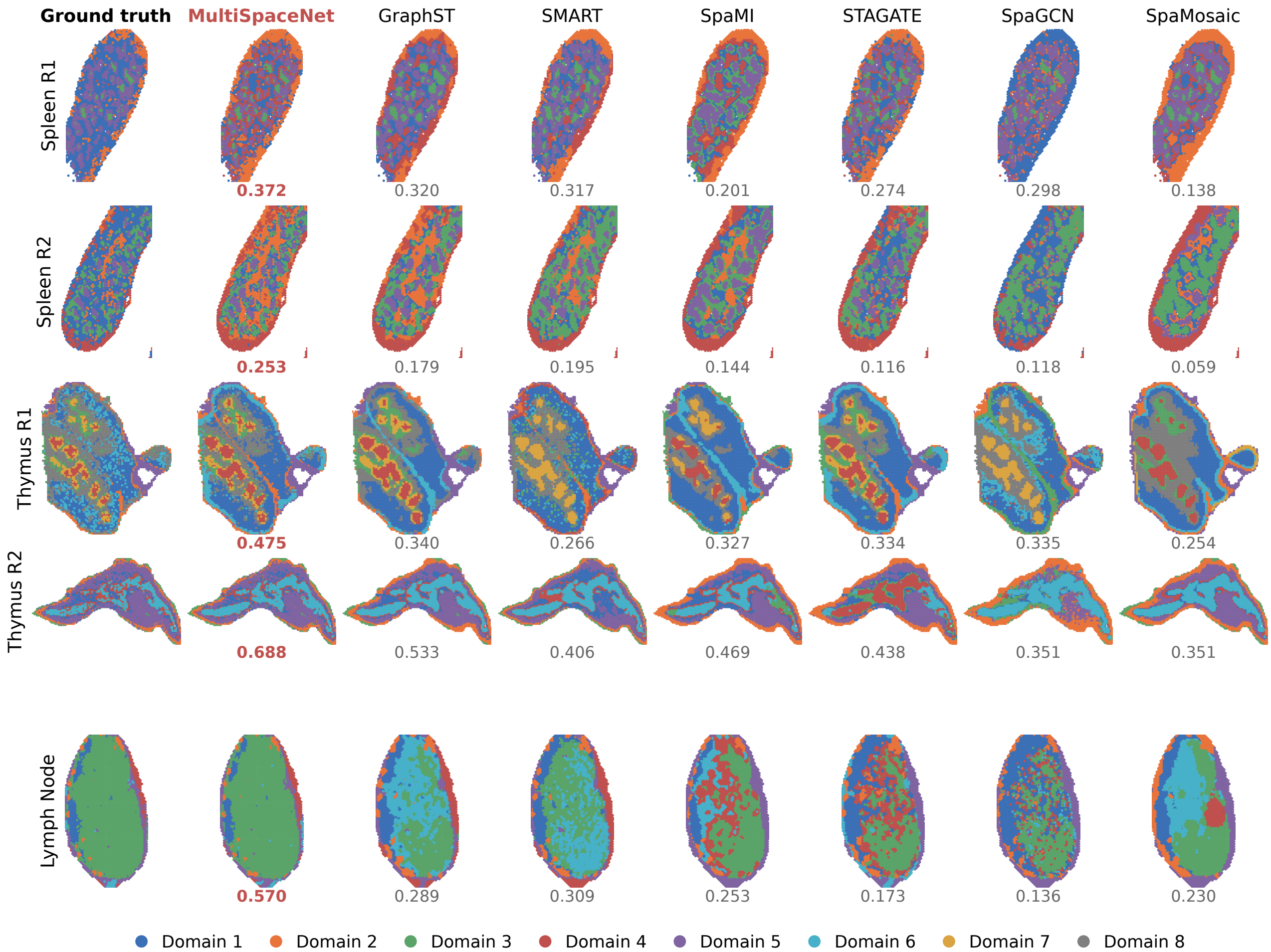

Fig. S1. Selected spatial domain maps on all five datasets. Rows are datasets; columns are the ground-truth annotation, MultiSpaceNet, and six baselines, ordered by mean ARI across datasets. SpatialGlue, CoMo, SpaMFG and ARISE are shown alongside these methods on the Human Lymph Node in main-text Fig. 2; the quantitative table, rather than this selected visual panel, defines the full comparator set. Baseline maps are obtained by clustering the stored embeddings with the same unified pipeline used throughout (L2 → PCA dimension capped at 20 → `mclust` EEE, seed 2020). The number beneath each map is the multi-seed mean ARI from Table 2; maps show a representative seed (for the bimodal Human Lymph Node, a seed from its upper mode; scores quoted are multi-seed means). Predicted cluster labels are matched to the ground-truth domains by maximum overlap so that a given domain keeps the same colour in every panel; because the ARI is invariant to label permutation this affects only the colouring, never the scores.

Table S1. Single a-priori training configuration used for all five datasets. All models use hidden dimension 128, 2 HANConv layers with 4 attention heads, 1 SAGEConv layer, dropout 0.1, Adam with learning rate 10^{-3}, and cosine annealing to $T_{\max} = 1{,}000$.

Dataset	Epochs	$T_{\max}$	w_{RNA}	w_{Protein}	w_{Cross}	Spatial k	Feature k
Spleen Rep1	1,000	1,000	1.0	3.0	1.0	3	20
Spleen Rep2	1,000	1,000	1.0	3.0	1.0	3	20
Thymus Rep1	1,000	1,000	1.0	3.0	1.0	6	20
Thymus Rep2	1,000	1,000	1.0	3.0	1.0	6	20
Lymph Node A1	1,000	1,000	1.0	3.0	1.0	6	20

Output projection

The refined representation is passed through a two-layer MLP output projection to obtain the final cell embedding:

$$\mathbf{z}_i \leftarrow \mathbf{W}_2\ \mathrm{ELU}\big(\mathbf{W}_1\,\mathbf{z}_i + \mathbf{b}_1\big) + \mathbf{b}_2, \tag{S7}$$

where $\mathbf{W}_1, \mathbf{W}_2 \in \mathbb{R}^{d\times d}$.

Learning rate schedule

We employ CosineAnnealingLR to gradually reduce the learning rate from $\eta_0 = 10^{-3}$ to $\eta_{\min} = 10^{-5}$:

$$\eta_t = \eta_{\min} + \frac{1}{2}\,(\eta_0 - \eta_{\min})\left(1 + \cos\frac{t\,\pi}{T}\right), \tag{S8}$$

where T is the total number of training epochs. This schedule is used throughout (with the fixed 1,000-epoch budget applied to every dataset); it was fixed a priori and not ablated.

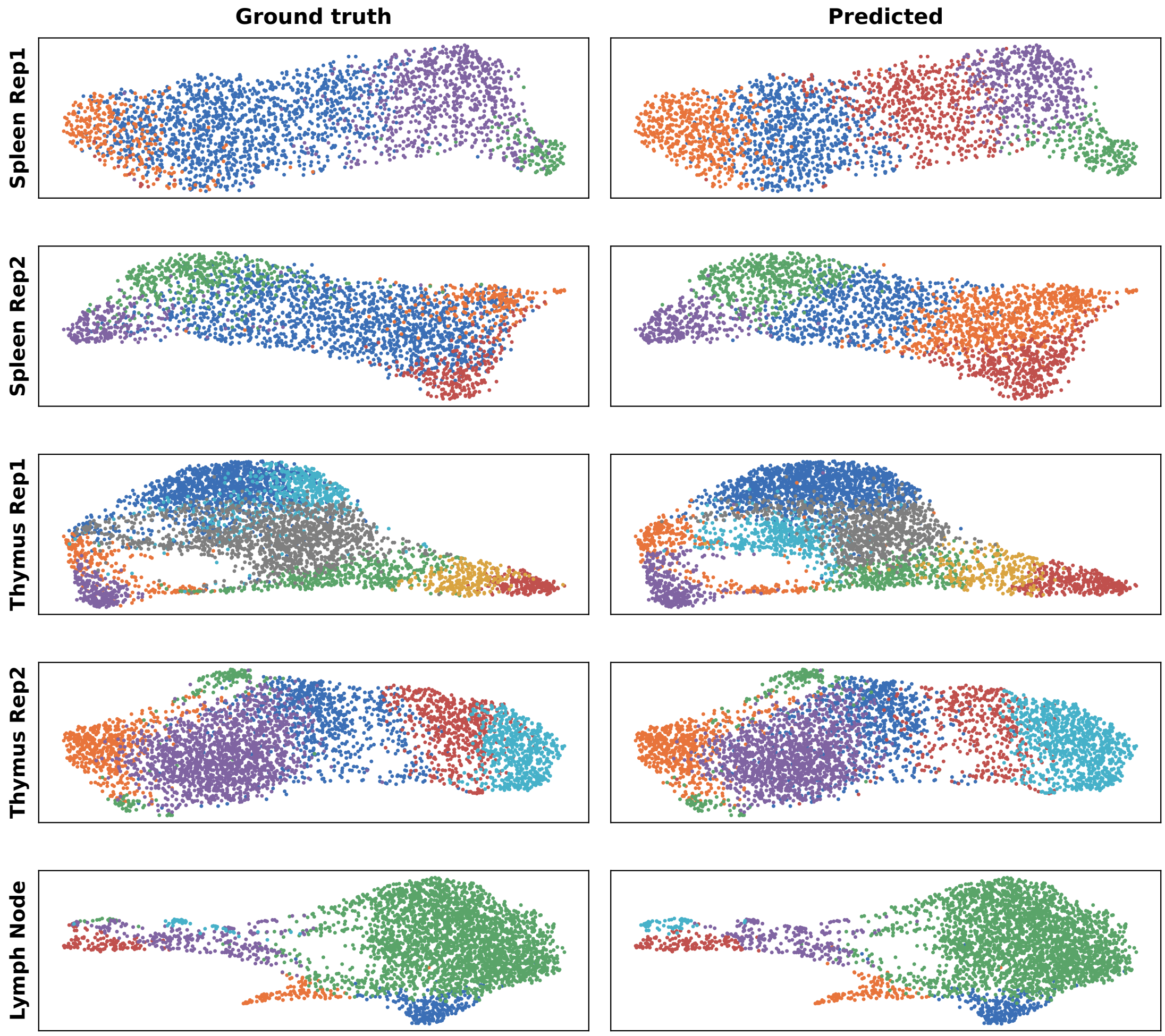

Fig. S2. UMAP embeddings of the MultiSpaceNet cell representations for all five datasets, coloured by ground-truth annotation (left) and predicted cluster (right). The learned representations form contiguous structure broadly consistent with the annotated domains, with residual overlap in line with the reported ARI values.

Training-loss convergence under the a-priori protocol

The fixed 1,000-epoch budget was set from the unsupervised training loss rather than from the evaluation metric, so that no per-dataset choice is informed by ARI. Figure S6 shows the self-supervised loss for all five datasets, normalised so that the curves are comparable. On every dataset the loss is within 1% of its final value by epoch 816–843, and over the final 100 epochs it changes by less than 0.7%; training is therefore flat well before the budget ends, and the same budget is adequate for all five datasets without tuning.

Recommended defaults and adapting to new data

The configuration in Table S1 is intended to be used as-is; only two settings carry any dataset dependence, and both follow platform conventions rather than tuning on the evaluation metric. The spatial neighbour count k_s reflects array geometry: we use $k_s = 3$ for the lower-density SPOTS arrays and $k_s = 6$ for the higher-resolution Stereo-CITE-seq and Visium CITE-seq data. The protein loss weight $w_p = 3$ corrects an imbalance of scale rather than of dimensionality: both modalities enter the encoders as principal components of the same dimension, but across these datasets the RNA block carries 5.6–47.7× the total variance of the protein block, so an unweighted objective is dominated by RNA. For a panel whose variance is closer to that of the RNA block, a value nearer $w_p = 1$ is a reasonable starting point, although we keep $w_p = 3$ fixed across all five datasets here; the sensitivity of both axes to this setting is reported in Table S21. All remaining settings—hidden dimension 128, two 4-head HANConv layers, one SAGEConv layer, $k_f = 20$, learning rate 10^{-3} and the fixed 1,000-epoch budget—are held constant and require no per-dataset adjustment.

Statistical Significance of ARI Improvements

To quantify the significance of MultiSpaceNet's performance gains under the a-priori protocol, we conducted paired tests between MultiSpaceNet (a-priori training) and SpatialGlue (unified L2 clustering), each over 10 random seeds, both scored by the identical pipeline. Table S2 reports the mean ARI difference, the one-sided paired Wilcoxon signed-rank p-value, and Cohen's d effect size for each dataset. All five comparisons are significant (Wilcoxon $p \leq 0.014$) with large effect sizes ($d \geq 0.99$), confirming the improvements are statistically significant and practically meaningful.

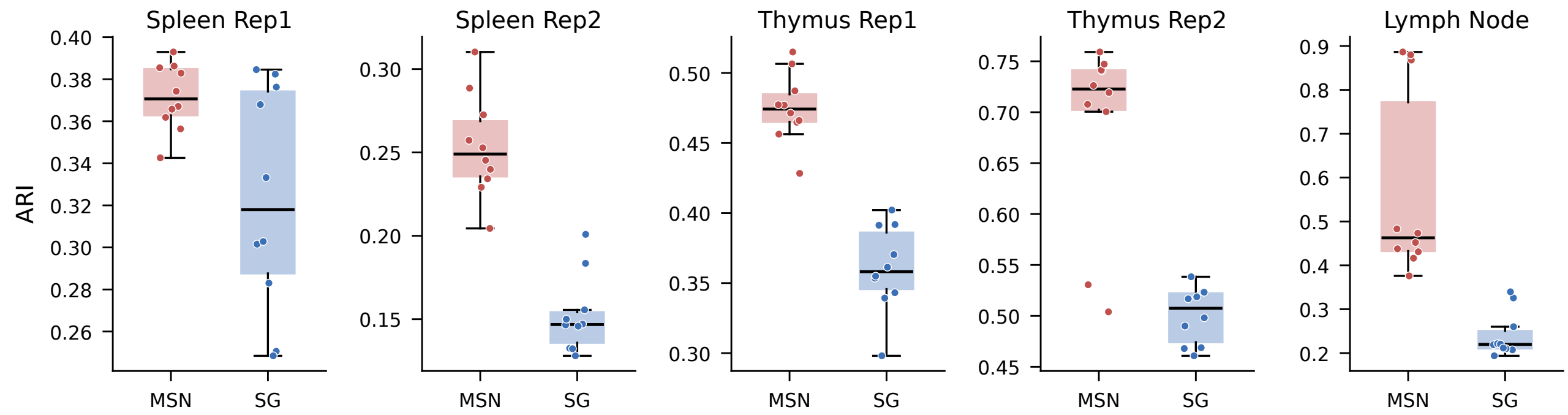

Fig. S3. ARI over 10 random seeds (box plus per-seed strip) for MultiSpaceNet (red) versus SpatialGlue (blue) under the unified clustering protocol, per dataset. The four mouse datasets show narrow seed-to-seed spread; the Human Lymph Node shows the bimodal seed behaviour discussed in the main text.

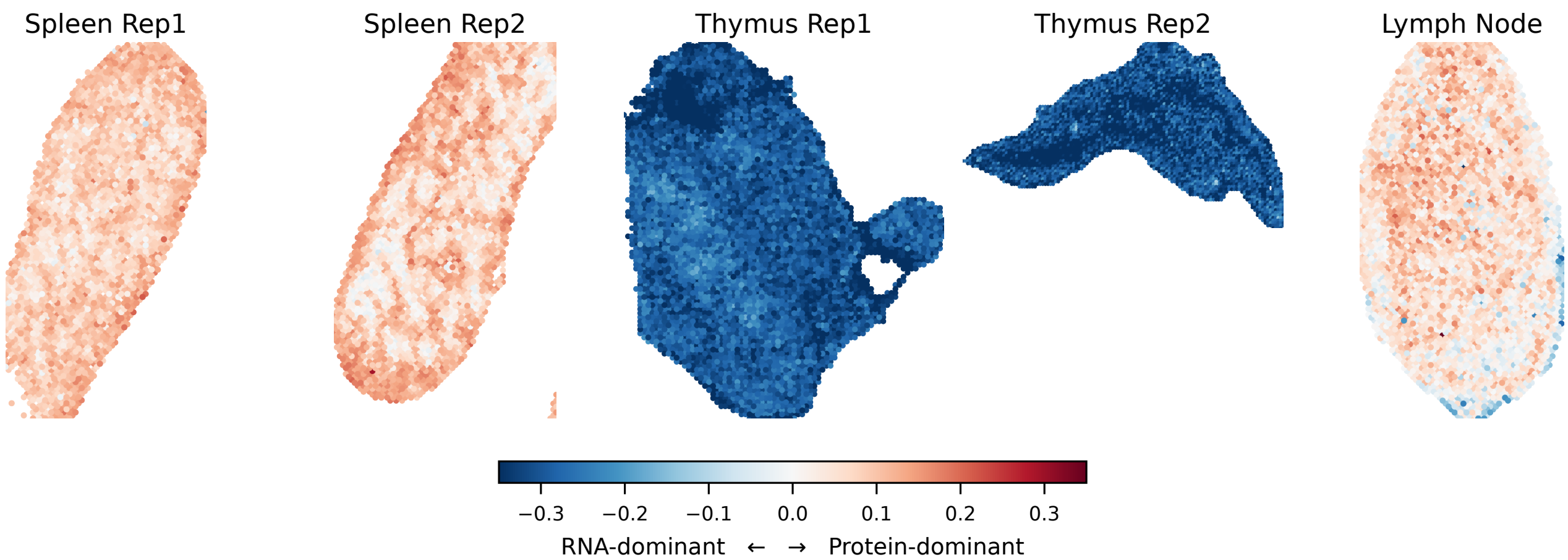

Fig. S4. Per-cell modality-dominance maps (protein minus RNA attention) for all five datasets. Blue indicates RNA-dominant and red protein-dominant cells. The thymus datasets are strongly RNA-dominant; the spleen and lymph node are near-balanced but retain fine-grained, spatially autocorrelated structure (Moran's I significant on every dataset). Maps are shown on a fixed scale, so the near-balanced datasets appear pale; the coherent structure is quantified by Moran's I (main-text Results, "Modality attention interpretation").

Per-Protein Cross-modal Prediction Results

Tables S3–S5 provide the complete per-protein Pearson correlation (r) for cross-modal protein expression prediction using Ridge regression from RNA embeddings. We compare MultiSpaceNet embeddings against SpatialGlue RNA_latent embeddings under identical 5-fold cross-validation. Bold values indicate the higher r for each protein. Overall, under the leakage-free protocol MultiSpaceNet wins on 98 out of 110 signal-bearing proteins (89.1%) across the five datasets (near-zero-signal proteins, mostly on Thymus Rep1, are excluded).

Note on Mouse Thymus Rep1.
The Stereo-CITE-seq protein panel for Thymus Rep1 contains 51 antibodies, but only 18 exhibited meaningful signal ($r > 0.1$ for either method); the remaining 33 had near-zero correlations for both methods, likely due to low expression or high technical noise. Under the leakage-free protocol MultiSpaceNet wins on

Table S2. Statistical significance of MultiSpaceNet's ARI improvements over SpatialGlue across five datasets, under the unified clustering protocol and a-priori training. Both methods were run with 10 random seeds; tests are paired seed-by-seed. Δ: mean ARI difference; Wilcoxon p: one-sided paired Wilcoxon signed-rank test; d: Cohen's d effect size.

Dataset	Δ ARI	Wilcoxon p	Cohen's d
Spleen Rep1	+0.049	0.014^{*}	0.99
Spleen Rep2	+0.101	0.001^{***}	3.43
Thymus Rep1	+0.114	0.001^{***}	3.51
Thymus Rep2	+0.187	0.003^{**}	1.85
Lymph Node A1	+0.329	0.001^{***}	1.45

Significance levels: $^{*}\,p < 0.05$; $^{**}\,p < 0.01$; $^{***}\,p < 0.001$.

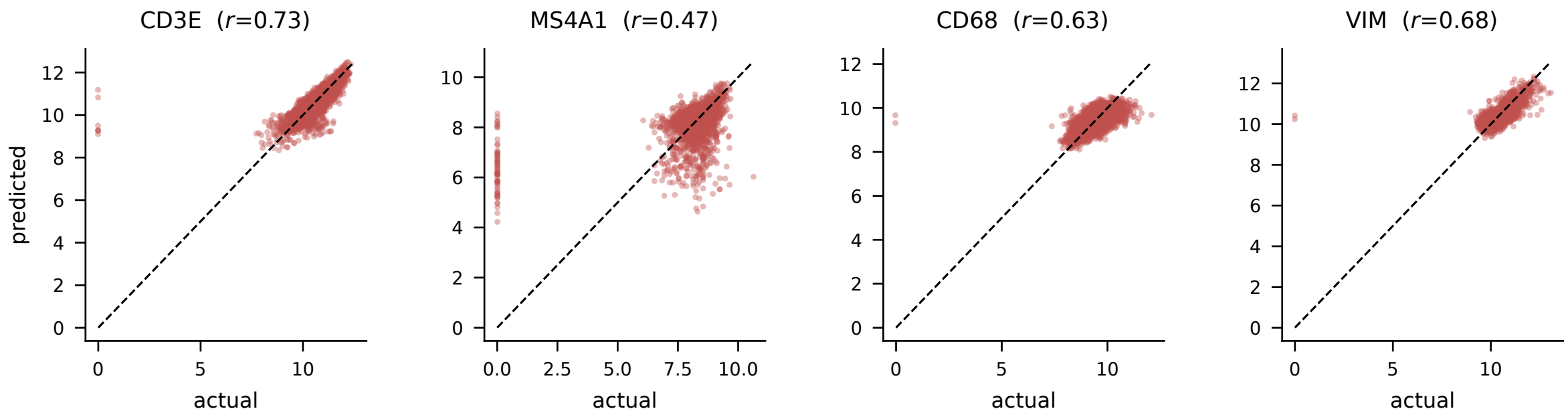

Fig. S5. Predicted versus actual protein expression (leakage-free inductive protocol) for four representative markers in the Human Lymph Node, with the identity line and Pearson r. The Ridge predictions from MultiSpaceNet's RNA embedding agree well with the measured protein, most strongly for CD3E and VIM.

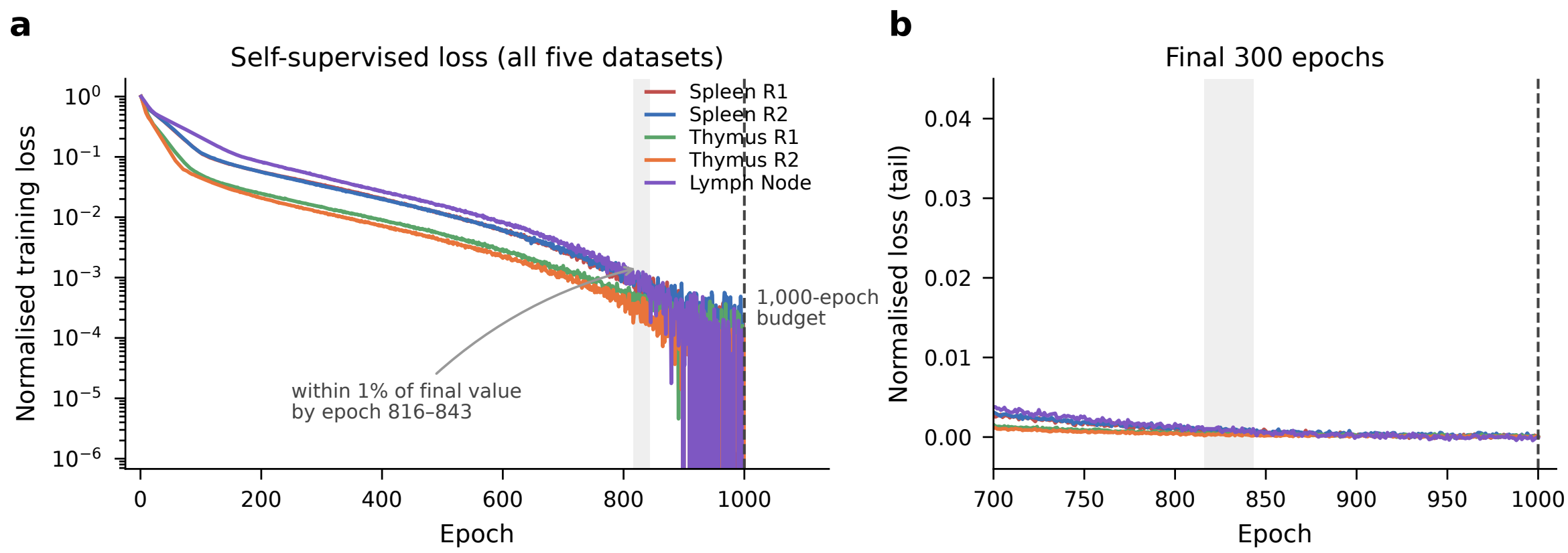

Fig. S6. Unsupervised training-loss convergence under the a-priori protocol. **(a)** Total self-supervised loss for all five datasets, normalised to 1 at initialisation and 0 at the final value, on a logarithmic axis. The shaded band marks the epochs at which the loss first comes within 1% of its final value (816–843); the dashed line is the fixed 1,000-epoch budget. **(b)** The same curves over the final 300 epochs, showing that training has flattened well before the budget ends. The budget was chosen from these curves, not from ARI.

15/18 of these signal-bearing proteins (main Table 3), with a mean r of 0.574 versus 0.549 for SpatialGlue.

Within-slide component and edge-type ablations

Tables S6 and S7 give the full within-slide ablations summarised in the main text, with mean $\pm$ SD over 10 seeds and a paired Wilcoxon test against the full model, matching the main benchmark. Under the a-priori protocol most differences fall within seed-to-seed variability: only three of the twenty-five component-ablation variant $\times$ dataset comparisons reach significance, and the edge-type table adds a single further significant effect. Removing HANConv significantly lowers ARI on Spleen Rep2 only; on Spleen Rep1 two simplifications (removing SAGEConv, or using three HANConv layers) slightly but significantly *raise* it. For the edge types, a single relation ties or exceeds the full three-relation graph on four of five datasets, the full graph being clearly best only on Spleen Rep2. We therefore present the full configuration as the formulation rather than as a set of individually necessary components.

Held-out protein-marker validation

The original post hoc marker panel overlapped the protein input. To make the validation input-independent, CD3, CD19, F4/80, CD169/Siglec-1 and CD31 were fixed as validation proteins and removed before protein normalisation, PCA and graph construction. They were absent from the RNA and protein reconstruction targets and from the RNA-to-protein loss. The remaining 16 proteins and the RNA measurements were used to train the otherwise unchanged model for ten seeds. After clustering, we quantified for each held-out marker the fraction of expression variance explained by the predicted domain (η^2). Table S8 reports effect sizes rather than forcing domains into a one-to-one anatomical matching. Permutation P values (999 label permutations and BH adjustment over the five markers) are supplied as a descriptive association check; because cells within a section are spatially dependent, the effect sizes and their seed stability carry the interpretation.

Joint-embedding visualisation for cross-sample integration

Figure S7 shows the joint embeddings behind the scIB metrics of the main text, projected to two dimensions with UMAP and coloured twice: by batch (replicate section), which shows how strongly each method mixes the sections, and by ground-truth spatial domain, which shows how much biological structure survives that mixing. The two views make the reported trade-off directly visible. SpaMosaic mixes the sections most aggressively but collapses the embedding into a thin manifold in which the

Table S3. Per-protein Pearson r for cross-modal protein prediction on the **Mouse Spleen** datasets (SPOTS, 21 proteins each), under the *leakage-free* inductive 5-fold protocol. MSN: MultiSpaceNet leakage-free RNA embedding; SG: SpatialGlue RNA_latent. Bold indicates the higher r. MultiSpaceNet wins on 19/21 (Rep1) and 20/21 (Rep2) proteins.

	**Spleen Rep1**		**Spleen Rep2**	
Protein	MSN r	SG r	MSN r	SG r
CD29	**0.693**	0.642	**0.680**	0.578
CD3	**0.718**	0.622	**0.773**	0.662
F4/80	0.814	**0.821**	**0.782**	0.773
CD169/Siglec	**0.667**	0.646	**0.671**	0.612
CD4	**0.731**	0.636	**0.757**	0.657
CD19	**0.729**	0.671	**0.770**	0.652
CD105	**0.577**	0.514	**0.629**	0.494
CD8	**0.626**	0.492	**0.708**	0.556
B220/CD45R	**0.738**	0.666	**0.762**	0.632
Ly6C	**0.426**	0.361	**0.488**	0.404
CD31	**0.548**	0.472	**0.672**	0.523
CD68	**0.707**	0.679	**0.706**	0.618
EpCAM	**0.317**	0.294	**0.360**	0.327
CD163	0.760	**0.787**	0.741	**0.753**
Ly6G	**0.440**	0.374	**0.522**	0.426
CD38	**0.645**	0.509	**0.731**	0.502
IgM	**0.486**	0.395	**0.542**	0.429
IgD	**0.727**	0.627	**0.802**	0.608
MAdCAM1	**0.401**	0.343	**0.492**	0.417
CD20	**0.402**	0.362	**0.460**	0.382
CD11b	**0.528**	0.464	**0.624**	0.479
**Mean**	**0.604**	0.542	**0.651**	0.547
**Win rate**	19/21 (90.5%)		20/21 (95.2%)	

Table S4. Per-protein Pearson r for cross-modal protein prediction on the **Mouse Thymus Rep2** dataset (Stereo-CITE-seq, 19 proteins), under the *leakage-free* inductive protocol. Bold indicates the higher r. MultiSpaceNet wins on 19/19 proteins.

Protein	MSN r	SG r
CD5	**0.735**	0.664
CD68	**0.641**	0.592
CD4	**0.764**	0.684
CD29	**0.509**	0.337
CD8a	**0.834**	0.770
CD3	**0.642**	0.483
CD44	**0.841**	0.793
CD27	**0.801**	0.721
CD90.2	**0.510**	0.353
CD11c	**0.846**	0.835
CD31	**0.534**	0.407
F4/80	**0.625**	0.482
B220/CD45R	**0.493**	0.378
IgG2a (Rat)	**0.525**	0.373
CD11b	**0.431**	0.361
CD19	**0.440**	0.336
IgG2a (Mouse)	**0.425**	0.302
Siglec-H	**0.372**	0.258
CD169/Siglec-1	**0.406**	0.379
**Mean**	**0.599**	0.500
**Win rate**	19/19 (100.0%)	

domains are no longer separated, consistent with its lowest bio-conservation on every scenario. Without integration the sections remain partly separated. MultiSpaceNet mixes the sections only moderately while keeping the domain structure that the dedicated integrators erode, which is the behaviour the bio-conservation scores quantify. Coordinates are from seed 0; the quantitative comparison over 5 seeds is in the main text and Supplementary Table S9.

Cross-sample integration: full scIB metrics

Table S9 gives the complete scIB metrics behind the trade-off shown in main-text Fig. 5: the batch-removal and biological-conservation means and the bio-ARI, for every method and scenario.

Missing-modality probe: modality contribution to clustering

Table S10 reports spatial-domain ARI when one modality is dropped at inference, over 5 seeds. It supports the honest

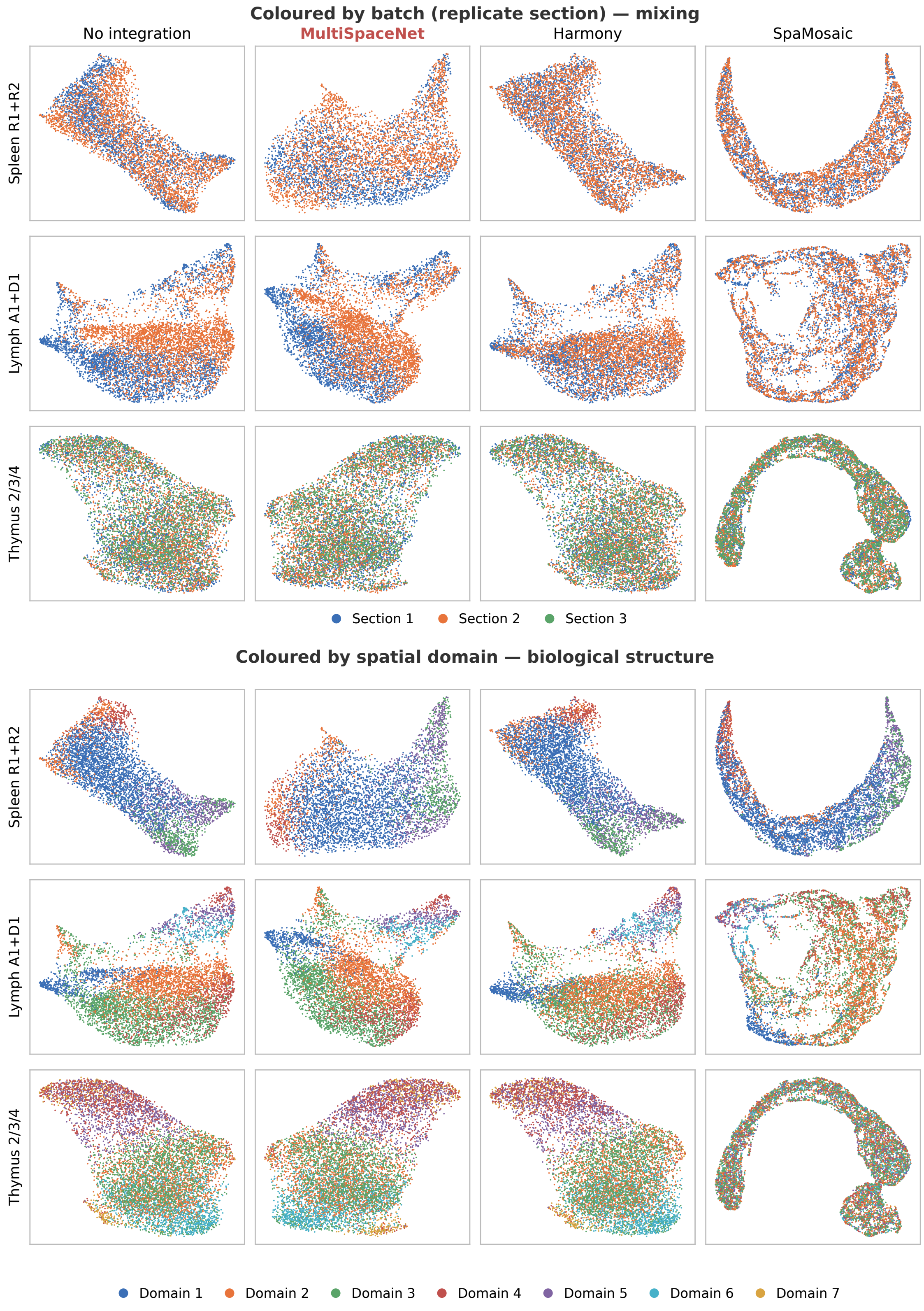

Fig. S7. UMAP of the joint embeddings for the three cross-sample scenarios (rows) and four methods (columns). Top block: cells coloured by batch, i.e. by replicate section. Bottom block: the same embeddings coloured by ground-truth spatial domain. Strong batch mixing with preserved domain separation is the desirable outcome; SpaMosaic achieves the former at the cost of the latter, whereas MultiSpaceNet retains domain structure under moderate mixing.

Table S5. Per-protein Pearson r for cross-modal protein prediction on the **Human Lymph Node A1** dataset (10x Visium CITE-seq, 31 proteins), under the *leakage-free* inductive protocol. Bold indicates the higher r. MultiSpaceNet wins on 25/31 proteins; on this dataset the embedding is competitive with but does not exceed the raw-RNA baseline (main text).

Protein	MSN r	SG r
ACTA2	**0.734**	0.710
CD3E	**0.733**	0.708
VIM	**0.683**	0.681
CD68	**0.628**	0.625
HLA-DRA	**0.576**	0.540
CD8A	**0.550**	0.538
BCL2	**0.548**	0.522
PAX5	**0.546**	0.497
CCR7	**0.517**	0.482
CXCR5	**0.515**	0.449
CD4	**0.483**	0.469
MS4A1	**0.468**	0.368
CD40	**0.463**	0.443
ITGAM	**0.460**	0.410
PTPRC-1	**0.452**	0.419
ITGAX	**0.447**	0.420
PTPRC	**0.431**	0.394
CR2	**0.419**	0.405
CEACAM8	**0.410**	0.395
PECAM1	0.392	**0.398**
PDCD1	**0.370**	0.370
FCGR3A	0.357	**0.391**
SDC1	**0.352**	0.272
CD163	0.344	**0.355**
CD274	**0.324**	0.295
KRT5	0.292	**0.304**
CD27	**0.279**	0.274
EPCAM	**0.267**	0.259
PCNA	**0.264**	0.255
CD19	0.152	**0.169**
CD14	0.117	**0.163**
**Mean**	**0.438**	0.419
**Win rate**	25/31 (80.6%)	

Table S6. Component ablation (mean ± SD ARI over 10 seeds, a-priori protocol). Paired Wilcoxon vs the full model: $^{*}p < 0.05$, $^{**}p < 0.01$. The table is grouped explicitly: the upper block removes a component, whereas the lower block varies the HANConv depth hyperparameter around the default of two layers and is a sensitivity check rather than an ablation; we state this grouping so the two kinds of variant are not read as one six-way comparison. The full-model row is the Table 2 configuration re-run jointly with the variants; its mean differs from Table 2 by ≤ 0.06 (random-seed variation, largest on the bimodal Lymph Node).

Variant	Spleen R1	Spleen R2	Thymus R1	Thymus R2	Lymph Node	Mean
Component removals						
Full model	0.373 ± 0.016	0.264 ± 0.021	0.475 ± 0.023	0.711 ± 0.079	0.519 ± 0.182	0.468
w/o HANConv	0.380 ± 0.016	$0.214 \pm 0.016^{**}$	0.484 ± 0.036	0.697 ± 0.024	0.541 ± 0.176	0.463
w/o SAGEConv	$0.404 \pm 0.039^{*}$	0.270 ± 0.026	0.498 ± 0.031	0.698 ± 0.086	0.442 ± 0.087	0.462
w/o Cross-modal	0.381 ± 0.021	0.262 ± 0.029	0.482 ± 0.021	0.706 ± 0.068	0.559 ± 0.213	0.478
Network-depth settings (hyperparameter sensitivity, not component removals)						
1 HANConv layer	0.385 ± 0.014	0.243 ± 0.015	0.497 ± 0.024	0.675 ± 0.079	0.612 ± 0.217	0.482
3 HANConv layers	$0.413 \pm 0.040^{*}$	0.268 ± 0.031	0.483 ± 0.025	0.681 ± 0.087	0.606 ± 0.225	0.490

Table S7. Edge-type ablation (mean ± SD ARI over 10 seeds, a-priori protocol). Paired Wilcoxon vs the full three-relation graph: $^{*}p < 0.05$, $^{**}p < 0.01$.

Variant	Spleen R1	Spleen R2	Thymus R1	Thymus R2	Lymph Node	Mean
All 3 relations	0.373 ± 0.016	0.264 ± 0.021	0.475 ± 0.023	0.711 ± 0.079	0.519 ± 0.182	0.468
Spatial only	0.371 ± 0.020	$0.233 \pm 0.018^{**}$	0.481 ± 0.028	0.693 ± 0.072	0.523 ± 0.177	0.460
Feature only (RNA+Prot)	0.380 ± 0.016	0.257 ± 0.015	0.470 ± 0.017	0.691 ± 0.069	0.572 ± 0.212	0.474
RNA similarity only	0.388 ± 0.023	0.248 ± 0.021	0.467 ± 0.031	0.715 ± 0.029	0.520 ± 0.183	0.468
Protein similarity only	0.371 ± 0.011	0.258 ± 0.020	0.476 ± 0.020	0.696 ± 0.062	0.464 ± 0.170	0.453

statement in the main text that, for domain identification, the second modality does not help on most datasets: RNA alone matches or exceeds the fused model on three of five datasets, and is also ahead on the five-dataset mean (0.484 versus 0.475, and 0.485 versus 0.458 once the bimodal Lymph Node is excluded). For spatial-domain identification specifically, the

Table S8. Input-independent biological validation on Mouse Spleen Rep1. Each listed protein was withheld from all model inputs, graphs and losses. Effect size is the fraction of held-out marker variance explained by the predicted domain, mean±SD over ten training seeds. The final column counts seeds with BH-adjusted permutation $q < 0.05$; it is descriptive because cells are not independent biological replicates. The held-out-input model's domain ARI is 0.414 ± 0.030.

Held-out marker	Pre-specified biological read-out	η^2	$q < 0.05$ seeds
CD3	White-pulp T zone	0.364 ± 0.017	10/10
CD19	White-pulp B follicle	0.370 ± 0.016	10/10
F4/80	Red-pulp macrophages	0.607 ± 0.015	10/10
CD169/Siglec-1	Marginal-zone macrophages	0.356 ± 0.032	10/10
CD31	Vascular/stromal pattern	0.046 ± 0.014	10/10

Table S9. Cross-sample integration (scIB metrics, mean over 5 seeds; higher is better). "batch" and "bio" are the mean of the batch-removal and bio-conservation sub-metrics; "overall" is the standard scIB composite $0.6 \times \text{bio} + 0.4 \times \text{batch}$; bio-ARI is the ARI component of bio, plotted in Fig. 5. Bold: best per scenario for each column. MultiSpaceNet has the highest bio-conservation of the four methods in every scenario, but because it performs essentially no batch removal (its batch score is at or below no-integration on two of three scenarios) the dedicated integrators Harmony and SpaMosaic lead on the batch-weighted composite; this is consistent with our positioning of MultiSpaceNet as a biology-preserving method rather than a batch-correction tool. The bio advantage over no-integration is small but consistent on the mean (positive in every scenario; paired t-test $p \leq 0.048$; the $n = 5$ Wilcoxon cannot fall below 0.0625), holding on 14 of the 15 individual seed runs (the exception is one lymph-node seed).

Scenario	Method	batch	bio	overall	bio-ARI
Spleen R1+R2	No integration	0.405	0.581	0.511	0.206
	**MultiSpaceNet**	0.411	**0.596**	0.522	**0.273**
	Harmony	0.786	0.570	**0.657**	0.211
	SpaMosaic	**0.818**	0.499	0.627	0.074
Lymph A1+D1	No integration	0.353	0.560	0.478	0.163
	**MultiSpaceNet**	0.343	**0.581**	0.486	**0.207**
	Harmony	0.640	0.538	0.579	0.149
	SpaMosaic	**0.827**	0.529	**0.648**	0.135
Thymus 2/3/4	No integration	0.681	0.536	0.594	0.202
	**MultiSpaceNet**	0.670	**0.544**	0.594	**0.214**
	Harmony	**0.852**	0.533	**0.661**	0.202
	SpaMosaic	0.789	0.513	0.623	0.191

second modality therefore does not pay for itself on these benchmarks. We report this because it bounds what the paired formulation can be claimed for: its supported value here is the joint representation and the conditional cross-sample result, not the clustering score. It beats the fused model significantly on Spleen Rep1 (+0.125, paired t-test $p = 0.001$), while protein adds value only on Thymus Rep2 and the Human Lymph Node (neither significant). This is why we do not claim that fusion improves clustering, and instead justify the paired formulation by the balanced joint representation (Table S16, with the untrained control in Table S18); the attention map is a descriptive attribution only, and the cross-modal read-out is reported as a practical imputation route. Values use seeds independent of the main benchmark, so "both" differs slightly from Table 2.

Edge-type ablation in the cross-sample integration regime

The within-slide edge-type ablation (Table S7) shows that the heterogeneous three-relation graph is competitive but not uniquely necessary. To check that this also holds for the integration task, we repeated the edge-type ablation on the three cross-sample scenarios, scoring biological conservation (ARI) for the full graph and for each single-relation variant (Table S11). In every scenario the best single-relation variant ties or exceeds the full graph, and the difference is not statistically significant, consistent with presenting the heterogeneous construction as a unified design choice rather than a necessary one.

Per-cell versus global modality fusion

The cell-level attention module learns a per-cell weighting of the RNA and protein branches. We test both whether that variation contributes to clustering and whether it responds to a known local loss of modality quality. Neither control supports a causal interpretation of the weights; we therefore report them only as descriptive model attributions.

Per-method protocol and hyperparameter provenance

Table S13 summarises, for every compared method, the input it consumes, the number of random seeds, and the source of its hyperparameters. All method outputs are scored through the identical downstream clustering pipeline, but each method retains its own published preprocessing and training procedure. MultiSpaceNet is held to the most constrained protocol (one dataset-independent configuration, no per-dataset selection on ARI); among the recent multi-omics baselines, SpaMI is the only method whose public code selects hyperparameters by dataset name (a tuned configuration for Mouse Thymus and a generic default otherwise), and for the three datasets that fall to its generic default (both spleen replicates and the lymph node) we additionally performed a small disclosed sweep, taking the best setting for the baseline. CoMo and SpatialCOC were instead run for the full schedules in their public tutorials, without a dataset-specific ARI sweep. ARISE [5] uses its official preprocessing, graph builder, model, loss and 350-epoch schedule for five seeds. Its released helper evaluates ground-truth ARI every epoch and returns the best-ARI embedding; to prevent label leakage, our common and native checks both use the final-epoch embedding and labels are consulted only afterwards. SpaMFG's released script has fixed spectral/MOFA

Table S10. Missing-modality probe on all five datasets: spatial-domain ARI (mean ± SD over 5 seeds) using both modalities, RNA only, or protein only. Δ is RNA-only minus both; bold marks Δ significant at $p < 0.05$ (paired t-test). RNA alone matches or exceeds the fused model on three of five datasets.

Dataset	Both modalities	RNA only	Protein only	Δ (RNA−both)
Spleen Rep1	0.368 ± 0.017	0.493 ± 0.020	0.200 ± 0.013	**+0.125**
Spleen Rep2	0.266 ± 0.013	0.272 ± 0.020	0.164 ± 0.006	+0.006
Thymus Rep1	0.471 ± 0.027	0.505 ± 0.063	0.215 ± 0.025	+0.034
Thymus Rep2	0.728 ± 0.020	0.670 ± 0.079	0.237 ± 0.028	−0.058
Lymph Node	0.542 ± 0.186	0.478 ± 0.213	0.324 ± 0.119	−0.064
Mean	0.475	0.484	0.228	+0.009
Mean, excl. Lymph Node	0.458	0.485	0.204	+0.027

Table S11. Edge-type ablation in the *cross-sample integration* regime (bio-conservation ARI, mean ± SD over 5 seeds). For each scenario the best single-relation variant ties or exceeds the full three-relation graph, and the difference (full vs. best single relation, paired Wilcoxon) is not significant. This complements the within-slide edge-type ablation (Table S7) and supports presenting the heterogeneous graph as a unified rather than necessary design. The full-graph row is the same configuration as MultiSpaceNet in the integration table (Table S9); the two 5-seed runs agree within seed noise (≤ 0.01).

Scenario	Edge configuration	bio-ARI	p
Spleen R1+R2	Full graph (3 relations)	0.273 ± 0.011	0.62
	Spatial only	0.248 ± 0.019	
	RNA-sim only	0.281 ± 0.030	
	Protein-sim only	0.273 ± 0.022	
Lymph A1+D1	Full graph (3 relations)	0.217 ± 0.012	0.44
	Spatial only	0.217 ± 0.013	
	RNA-sim only	0.214 ± 0.018	
	Protein-sim only	0.227 ± 0.020	
Thymus 2/3/4	Full graph (3 relations)	0.206 ± 0.016	0.81
	Spatial only	0.211 ± 0.018	
	RNA-sim only	0.196 ± 0.026	
	Protein-sim only	0.204 ± 0.017	

Table S12. Per-cell modality attention versus a dataset-global modality weight (spatial-domain ARI, mean over 5 seeds). Replacing the learned per-cell weights at inference with their dataset-global mean produces no significant change on any dataset (pooled paired Wilcoxon $p = 0.60$; on the bimodal Lymph Node the point estimate favours the global weight by 0.23; we do not discount it, and note only that this is the dataset whose bimodal seed distribution makes single point estimates unreliable for every arm), so the per-cell map is reported as an interpretability read-out rather than a performance lever. The per-cell column is the default MultiSpaceNet under this 5-seed experiment and matches the 10-seed main-table ARIs within seed variability (the Lymph Node difference reflects the bimodal seed behaviour, Fig. S3). The per-cell protein-attention map is nonetheless significantly spatially autocorrelated on every dataset (Moran's I; permutation $p < 0.05$ in every run).

Dataset	Per-cell	Global	Δ	p	Moran's I
Spleen Rep1	0.370	0.377	-0.006	0.06	0.38–0.48
Spleen Rep2	0.278	0.264	+0.014	0.31	0.48–0.59
Thymus Rep1	0.474	0.472	+0.002	0.81	0.40–0.54
Thymus Rep2	0.719	0.706	+0.013	0.19	0.31–0.46
Lymph Node	0.483	0.709	-0.227	0.12	0.30–0.40

seeds and settings, so it is run once on each dataset rather than presenting identical repeats as variability; its common-evaluator failures on the two thymus embeddings are retained. HEIST is discussed in the Introduction but excluded from this paired-matrix benchmark because its released input route requires preprocessed cell graphs and per-cell gene-regulatory-network graph objects rather than paired RNA/ADT matrices; no direct documented path produces a comparable joint embedding.

Native-protocol cross-check for recent multi-omics baselines

Because we score all methods with our own unified clustering pipeline, we additionally re-scored recent multi-omics baselines with their *own* native clustering backends (SMART with `mclust` EEE; SpaMI with Leiden at resolution 0.55; SpaMFG with Leiden at its released resolution 0.3; ARISE with KMeans at the annotated K), to check the ranking under the methods' own read-outs (Table S14). For ARISE, KMeans is applied to the same label-free final-epoch embedding rather than to a ground-truth-selected epoch. MultiSpaceNet remains ahead on every dataset. This table is especially important for SpaMFG: it gives complete native coverage while keeping the two failed common-evaluator cells blank in the main table.

Table S13. Per-method evaluation protocol and hyperparameter provenance. All methods are scored through the identical clustering pipeline (L2 → PCA dimension capped at 20 → `mclust` EEE, seed 2020) with the ground-truth cluster number; an embedding already at or below 20 dimensions is retained. "Input" indicates what each method consumes. Upstream preprocessing remains method-native, and ARI uses the annotated cells returned by that pipeline; retained-cell counts accompany every per-seed machine-readable result. MultiSpaceNet is held to the most constrained protocol (a single dataset-independent configuration, no per-dataset selection on ARI).

Method	Type	Input	Seeds	Hyperparameters
MultiSpaceNet	multi-omics	paired RNA+protein	10	Fixed a-priori config (1,000 ep, w_p=3); no per-dataset ARI tuning
SpatialGlue	multi-omics	paired RNA+protein	10	Authors' default settings
SMART (2026)	multi-omics	paired RNA+protein	5	Authors' default settings (native backend = mclust EEE)
SpaMI (2025)	multi-omics	paired RNA+protein	5	Native tuned config on Mouse_Thymus; generic default elsewhere + disclosed light sweep
SpaMosaic (2026)	mosaic integrator	paired RNA+protein	5	Authors' demo settings (repurposed for single-slide)
CoMo (2026)	multi-omics	paired RNA+protein	5	Official tutorial staged schedule (300-epoch reconstruction pretraining; 600 total epochs)
ARISE (2026)[§]	multi-omics	paired RNA+protein	5	Official preprocessing/model/loss and 350 epochs; final embedding scored without label-based epoch selection
SpatialCOC (2026)[†]	multi-omics	paired RNA+protein	5	Official SPOTS/Stereo-CITE-seq tutorials (SCM 500/500 or 1,500/500 epochs; COC 150 epochs)
SpaMFG (2026)[‡]	multi-omics	paired RNA+protein	1 (fixed)	Released feature-group/MOFA script: 10 groups, 50 neighbours/group, 2 factors/group; spectral seed 42, MOFA seed 1
GraphST	single-omics	concat. RNA+protein	10	Authors' default settings
SpaGCN	single-omics	concat. RNA+protein	10	Authors' default settings
STAGATE	single-omics	concat. RNA+protein	10	Authors' default settings

[§]The released ARISE helper selects its best epoch by ground-truth ARI. We retain the official 350-epoch budget but use the final-epoch embedding, so no label enters optimisation or model selection. [†]Evaluated on the four shared mouse datasets. Its official preprocessing does not accept the 10x Visium CITE-seq lymph-node format, so that entry is left blank rather than produced by an unpublished adaptation. [‡]Run on all five datasets. The unified mclust EEE evaluator succeeds on both spleens and the lymph node but returns no fitted model on the two thymus embeddings; those common-score entries are left blank and the released Leiden results are reported in Table S14.

Table S14. Native-protocol cross-check. SMART and SpaMI are scored with their own native clustering backends (SMART: `mclust` EEE; SpaMI: Leiden, resolution 0.55), mean ARI over 5 seeds. SpaMFG is scored with its released Leiden resolution 0.3 and fixed seeds (one deterministic run); its native Lymph Node score (0.318) closely reproduces the published value (0.316), providing an implementation check. ARISE uses KMeans at the annotated K on its final-epoch embedding (mean ± SD over 5 seeds); this intentionally replaces the released ground-truth-ARI best-epoch selection with a label-free fixed-epoch read-out. The MultiSpaceNet column is its main-benchmark result (10 seeds, unified evaluator). MultiSpaceNet remains ahead on all five datasets. SpaMFG's native scores are not substituted for the two unavailable common-evaluator entries in the main table.

Dataset	MultiSpaceNet	SMART	SpaMI	SpaMFG	ARISE
Spleen Rep1	**0.372**	0.263	0.182	0.134	0.266 ± 0.023
Spleen Rep2	**0.253**	0.175	0.126	0.055	0.089 ± 0.035
Thymus Rep1	**0.475**	0.283	0.268	0.186	0.259 ± 0.001
Thymus Rep2	**0.688**	0.423	0.329	0.304	0.297 ± 0.025
Lymph Node	**0.570**	0.286	0.152	0.318	0.337 ± 0.005

Table S15. Extended cross-modal protein-prediction baselines, under the identical leakage-free inductive 5-fold protocol, folds, and target as Table 3 (main text). Mean Pearson r over signal-bearing proteins against the measured protein (log-normalised). *Ridge* and *MLP* are a linear and a two-hidden-layer non-linear regressor on the raw RNA PCA features; *totalVI* and *sciPENN* are the two purpose-built generative CITE-seq protein-imputation methods, each trained per fold on the training cells only and used to impute protein for the held-out cells from RNA. MultiSpaceNet exceeds or matches every baseline on every dataset (a near-tie with the Ridge baseline on the lymph node, 0.438 vs. 0.437). **Caveat for a fair reading:** totalVI and sciPENN are optimised to output a *compositional, per-cell-normalised* protein rather than the measured log-count target scored here, so this table reflects accuracy at *recovering measured protein abundance* (the practical imputation use-case) rather than a like-for-like generative benchmark; the direct per-protein predictors (MultiSpaceNet, Ridge) lead on it, consistent with the established advantage of direct supervised prediction over amortised generative imputation for per-protein accuracy. totalVI is additionally numerically unstable on the high-count antibody panels (Thymus Rep2, Lymph Node, requiring gradient clipping) and diverges on the sparse 51-protein Thymus Rep1 panel (†), whereas sciPENN trains stably there (0.284)—so the Thymus Rep1 collapse is specific to totalVI, not generic to imputation.

Dataset	MultiSpaceNet	Ridge (raw RNA)	MLP (raw RNA)	totalVI	sciPENN
Spleen Rep1	**0.604**	0.562	0.501	0.316	0.378
Spleen Rep2	**0.651**	0.582	0.582	0.335	0.322
Thymus Rep1	**0.574**	0.550	0.541	−0.057[†]	0.284
Thymus Rep2	**0.599**	0.526	0.555	0.253	0.268
Lymph Node A1	**0.438**	0.437	0.340	0.279	0.364
Mean	**0.573**	0.531	0.504	0.225	0.323

Table S16. Cross-modal structure preservation for the spatial multi-omics methods with saved joint embeddings. Balance is $\min(J_{\text{RNA}}, J_{\text{prot}})$, where each J is the Jaccard overlap between the embedding kNN graph ($k = 30$) and the corresponding modality-only graph. Comparator cell sets match ours on Spleen Rep1, Spleen Rep2 and the Lymph Node; all method means are therefore calculated on that common three-dataset subset. The published comparator embeddings use different thymus cell sets, so those cells are left blank and no thymus comparison is made. On the matched subset MultiSpaceNet has balance 0.092, against SMART 0.063, SpatialGlue 0.048, SpaMI 0.036 and SpaMosaic 0.037. The variance-scaled concatenation is a diagnostic ceiling, not a learned competitor: it reaches 0.185 on the same subset but only 0.332 mean ARI on the five-dataset downstream benchmark. Thus balance is reported as a neighbourhood-retention diagnostic, not a utility ranking. Seed 0; Table S22 reports multiseed stability and coverage.

	Learned / naive representations						Diagnostic
Dataset	MultiSpaceNet	SpatialGlue§	SMART§	SpaMI§	SpaMosaic§	Concat.	Concat.‡
Spleen Rep1	**0.095**	0.034	0.057	0.025	0.026	0.054	0.195
Spleen Rep2	**0.091**	0.034	0.052	0.024	0.027	0.053	0.178
Thymus Rep1	0.045	—	—	—	—	0.034	0.170
Thymus Rep2	0.029	—	—	—	—	0.021	0.142
Lymph Node A1	**0.089**	0.078	0.079	0.059	0.058	0.068	0.182
Matched-3 mean	**0.092**	0.048§	0.063§	0.036§	0.037§	0.058	0.185

Table S17. Cross-modal protein prediction is not a well-posed task for the early-fusion integrators. Every entry is the mean Pearson r over signal-bearing proteins under the *identical* Ridge protocol and folds as Table 3. Two references bracket the scale: a Ridge on the RNA features, which has no access to protein at all, and a Ridge on the model's own protein input features, which is handed the target directly. SMART, SpaMI and SpaMosaic fuse the modalities at their input, so their joint embedding is a function of the measured protein; scoring them here recovers that input rather than predicting it, and SMART reaches 99.4% of the direct-access reference on the mean (0.8235/0.8287 at full precision; the table prints three decimals) (exceeding it on three datasets, since a learned non-linear function of the protein can carry more of the measured log-counts than 20 linear components of it). This is why the cross-modal comparison in Table 3 is restricted to modality-separable representations. Baseline embeddings are the same ones used for Table 2; mean over 5 seeds.

	No protein access	Early-fusion joint embedding			Direct protein access
Dataset	raw RNA (Ridge)	SpaMI	SpaMosaic	SMART	protein features (Ridge)
Spleen Rep1	0.562	0.591	0.662	0.888	0.788
Spleen Rep2	0.582	0.643	0.705	0.876	0.822
Thymus Rep1	0.550	0.669	0.756	0.812	0.980
Thymus Rep2	0.526	0.588	0.707	0.889	0.749
Lymph Node A1	0.437	0.488	0.523	0.652	0.805
Mean	0.531	0.596	0.671	0.823	0.829

Table S18. What training contributes, isolated on the three axes measured in this run. Each row compares the same architecture with the same initial weights on the same data under the fully a-priori protocol of Table 2 (fixed 1,000 epochs, $w_p = 3$); the only difference is whether the training loop is run. Domain-identification ARI and cross-modal structure preservation (balance $= \min(J_{\text{RNA}}, J_{\text{prot}})$, $k = 30$) improve on all five datasets. The untrained network's mean balance (0.038) sits near the bottom of the compared field—only SpaMI (0.036) and SpaMosaic (0.034) are lower (Table S16)—while the trained network's (0.071) is the highest mean of all methods, and reproduces the seed-0 values of Table S16 to ≤ 0.006 on every dataset. We also report the third axis the same run measured, the spatial autocorrelation of the per-cell protein-attention map, *including where it does not support us*: training raises Moran's I on all five datasets, but the untrained network already reaches 0.275 on the mean, so much of the map's spatial structure is inherited from the input features rather than created by training, and we claim only that the map is spatially structured, not that training produces that structure. Mean over 10 seeds per arm.

	Domain identification (ARI)		Structure preservation		Attention Moran's I	
Dataset	untrained	trained	untrained	trained	untrained	trained
Spleen Rep1	0.324	0.394	0.042	0.095	0.180	0.429
Spleen Rep2	0.173	0.264	0.041	0.091	0.217	0.494
Thymus Rep1	0.371	0.467	0.036	0.044	0.363	0.501
Thymus Rep2	0.406	0.707	0.022	0.027	0.287	0.353
Lymph Node A1	0.278	0.615	0.051	0.096	0.331	0.353
Mean	0.310	0.489	0.038	0.071	0.275	0.426

Table S19. Component ablation re-scored on two further axes beyond ARI. The ablation in Supplementary Tables S6 and S7 scores every variant by spatial-domain ARI; here the identical variants are scored by leakage-free cross-modal prediction (inductive 5-fold, mean Pearson r over signal-bearing proteins) and by structure-preservation balance ($\min(J_{\text{RNA}}, J_{\text{prot}})$, $k = 30$), mean over 3 seeds. Neither axis separates the variants: cross-modal r spans 0.579–0.580 on the mean and balance spans 0.069–0.073, with the full model not the highest on either. Removing the cross-modal decoder—the term that exists to create this ability—changes cross-modal r by 0.000. Taken with Table S18, the effect of training is real but is not attributable to the typed graph, the attention fusion, or any individual component; the three-relation construction is the formulation rather than a demonstrated source of the gain, as stated in the Conclusion.

	Cross-modal prediction (r, signal proteins)						Structure preservation (balance)					
Variant	Spleen R1	Spleen R2	Thymus R1	Thymus R2	Lymph	Mean	Spleen R1	Spleen R2	Thymus R1	Thymus R2	Lymph	Mean
Full model	0.606	0.653	0.601	0.595	0.440	0.579	0.085	0.089	0.045	0.028	0.110	0.071
w/o Cross-modal loss	0.605	0.651	0.601	0.595	0.442	0.579	0.085	0.088	0.045	0.028	0.110	0.071
w/o HANConv (0 layers)	0.607	0.655	0.604	0.596	0.440	0.580	0.082	0.081	0.045	0.028	0.117	0.071
w/o SAGEConv	0.602	0.653	0.603	0.598	0.438	0.579	0.087	0.090	0.045	0.027	0.114	0.073
Only spatial edges	0.606	0.652	0.602	0.595	0.441	0.579	0.083	0.085	0.044	0.028	0.109	0.070
Only RNA edges	0.606	0.653	0.603	0.596	0.442	0.580	0.083	0.084	0.044	0.028	0.106	0.069
Only protein edges	0.606	0.652	0.604	0.595	0.441	0.580	0.090	0.091	0.046	0.028	0.112	0.073

Table S20. Cross-sample biological conservation against published, matched-architecture, model-free and untrained comparators. Biological conservation (scIB bio-ARI) on the three within-platform replicate scenarios, five seeds each, every representation scored with identical metric code. The MultiSpaceNet row is this run's own re-evaluation; it reproduces Table S9 to within rounding (Spleen R1+R2 0.274 against 0.273, identical elsewhere), and every contrast in this table is computed within this run. The comparator set was extended at review in three ways: SpatialGlue, run on the same joint sections with its own preprocessing; the *matched no-graph autoencoder*, which shares this model's objective, encoders and attention fusion and differs only in that the typed graph and the SAGEConv layer are removed; and two model-free references. MultiSpaceNet is highest in every scenario against every comparator. Pooled over the fifteen individual seed runs (paired Wilcoxon): versus the matched no-graph autoencoder $\Delta = +0.044$, wins 13/15, $p = 0.0034$; versus SpatialGlue $\Delta = +0.059$, wins 13/15, $p = 0.0026$; versus naive concatenation $\Delta = +0.041$, wins 12/15, $p = 0.0020$; versus Harmony $\Delta = +0.044$, wins 12/15, $p = 0.0012$; versus variance-scaled concatenation $\Delta = +0.063$, wins 12/15, $p = 0.0034$; versus the untrained network $\Delta = +0.072$, wins 15/15, $p = 0.0001$. Two of these matter most. The untrained arm shares the architecture, the initialisation distribution, the preprocessing and the scoring code, so it isolates training; the no-graph arm shares the training and the objective, so it isolates the typed graph. On the within-slide axes the no-graph control is indistinguishable from the full model (Table S21); on this axis it is not, which is why we locate the contribution here. SMART and SpaMI are single-section methods whose released code we could not instantiate for the joint-section setting, and we state that rather than imply coverage we do not have. Harmony and SpaMosaic remain ahead on the batch-weighted scIB composite (Table S9); the quantity here is biological conservation.

Representation	Spleen R1+R2	Lymph A1+D1	Thymus 2/3/4	Mean
**MultiSpaceNet**	**0.274** $\pm$ 0.012	**0.207** $\pm$ 0.040	**0.214** $\pm$ 0.011	**0.232**
No-graph autoencoder (matched objective)	0.193 $\pm$ 0.011	0.186 $\pm$ 0.002	0.185 $\pm$ 0.014	0.188
SpatialGlue	0.176 $\pm$ 0.018	0.148 $\pm$ 0.032	0.194 $\pm$ 0.021	0.173
Harmony (dedicated batch corrector)	0.211 $\pm$ 0.003	0.149 $\pm$ 0.001	0.202 $\pm$ 0.004	0.187
Naive concatenation (no integration)	0.206 $\pm$ 0.000	0.163 $\pm$ 0.000	0.202 $\pm$ 0.000	0.190
Variance-scaled concatenation	0.127 $\pm$ 0.025	0.176 $\pm$ 0.001	0.202 $\pm$ 0.004	0.168
MultiSpaceNet, untrained	0.177 $\pm$ 0.029	0.130 $\pm$ 0.015	0.172 $\pm$ 0.016	0.160

Table S21. Sensitivity to the protein-loss weight, and a matched-objective control for the typed graph. Both analyses were requested at review and we report them at 10 seeds throughout, under the a-priori protocol of Table 2 (fixed 1,000 epochs). Left: spatial-domain ARI per dataset; right: mean cross-modal structure-preservation balance. The no-graph control shares the objective, the per-modality encoders and the attention fusion with MultiSpaceNet and differs only in that the typed graph and the SAGEConv layer are removed, so what separates the two is the graph itself. **(i)** The balance metric tracks w_p monotonically ($0.026 \rightarrow 0.044 \rightarrow 0.071$), because raising w_p trades RNA-neighbourhood retention for protein-neighbourhood retention and the balance is the smaller of the two; it is therefore a diagnostic of that trade rather than a ranking, and the field-leading value of Table S16 holds at the a-priori setting rather than in general. **(ii)** The typed graph is worth +0.026, +0.001 and −0.007 mean ARI at $w_p = 0$, 1 and 3 respectively. We note that at 3 seeds the $w_p = 1$ difference appeared to be +0.096; it does not survive 10 seeds, as the corresponding $w_p = 1$ advantage of the full model over $w_p = 3$ did not (Methods, "Evaluation pipeline"). We therefore retain the a-priori $w_p = 3$ rather than re-select on the metric, and we present the multi-relational graph as the formulation rather than a demonstrated source of the gain. *the configuration used throughout the paper. Its mean ARI here (0.464) differs from Table 2's 0.472 although both are 10 seeds of the same configuration: the runs are independent and the difference is carried almost entirely by the bimodal Lymph Node (Methods, "Evaluation pipeline"), whose seed distribution places its mean anywhere in 0.45–0.57 across our own repetitions. The same caveat applies to the no-graph column, and it is why we read the graph-versus-no-graph contrast from the five-dataset mean rather than from that dataset.

		Spatial-domain ARI						Balance
Model	w_p	Spleen R1	Spleen R2	Thymus R1	Thymus R2	Lymph Node	Mean	Mean
MultiSpaceNet	0	0.513	0.248	0.478	0.590	0.434	0.453	0.026
No-graph autoencoder	0	0.502	0.266	0.459	0.477	0.429	0.427	0.025
MultiSpaceNet	1	0.440	0.262	0.474	0.688	0.509	0.475	0.044
No-graph autoencoder	1	0.486	0.223	0.455	0.563	0.643	0.474	0.044
MultiSpaceNet*	3	0.387	0.264	0.480	0.678	0.511	0.464	0.071
No-graph autoencoder	3	0.401	0.192	0.422	0.608	0.733	0.471	0.065

Table S22. Seed stability of the structure-preservation metric. Table S16 reports one seed per method; here every method for which multiple saved embeddings exist is rescored over all of them, with identical metric code, so the dispersion of the ranking can be judged. Values are mean ± SD of the balance $\min(J_{\mathrm{RNA}}, J_{\mathrm{prot}})$, $k = 30$; the last column gives the number of seeds ("det." marks the two deterministic model-free references). Seed-to-seed dispersion is small for every method rescored here (SD ≤ 0.005 throughout, and ≤ 0.003 on every dataset except the Human Lymph Node, which is the highest-variance benchmark here as it is on ARI), so the ordering in Table S16 is stable. The strongest learned competitor is SMART; on the three datasets whose saved embeddings match our cell set, MultiSpaceNet averages 0.094 against SMART's 0.061 and SpatialGlue's 0.048, a margin an order of magnitude larger than the per-dataset dispersion. Coverage is not uniform: SMART, SpaMI and SpaMosaic supply saved embeddings whose cell sets match ours on three of the five datasets, so their means are over those three; the other methods cover all five. ‡the model-free variance-scaled concatenation of Table S16 is included as the ceiling of the metric, not as a competing representation—it attains the highest balance of anything measured while placing only mid-field on the downstream task (Results, "Cross-modal structure preservation").

Representation	Spleen R1	Spleen R2	Thymus R1	Thymus R2	Lymph Node	Mean	Seeds
**MultiSpaceNet**	**0.096** ± 0.000	**0.091** ± 0.002	**0.045** ± 0.001	**0.028** ± 0.001	**0.095** ± 0.005	**0.071**	3
SMART	0.053 ± 0.002	0.051 ± 0.003	—	—	0.079 ± 0.001	0.061	5
SpatialGlue	0.034 ± 0.001	0.034 ± 0.001	—	—	0.078 ± 0.004	0.048	5
GraphST	0.045 ± 0.000	0.041 ± 0.000	0.054 ± 0.000	0.037 ± 0.000	0.070 ± 0.000	0.049	10
STAGATE	0.033 ± 0.000	0.031 ± 0.001	0.051 ± 0.000	0.039 ± 0.000	0.055 ± 0.001	0.042	10
SpaGCN	0.031 ± 0.001	0.027 ± 0.004	0.044 ± 0.002	0.028 ± 0.002	0.040 ± 0.003	0.034	10
SpaMI	0.025 ± 0.000	0.023 ± 0.000	—	—	0.058 ± 0.001	0.035	5
SpaMosaic	0.026 ± 0.000	0.027 ± 0.000	—	—	0.058 ± 0.000	0.037	5
Naive concatenation	0.054 ± 0.000	0.053 ± 0.000	0.034 ± 0.000	0.021 ± 0.000	0.068 ± 0.000	0.046	det.
Variance-scaled concat.‡	0.195 ± 0.000	0.178 ± 0.000	0.170 ± 0.000	0.142 ± 0.000	0.182 ± 0.000	0.173	det.

Table S23. The two components of the structure-preservation metric, reported separately for every representation. Table S16 prints only the minimum of the two Jaccard overlaps; that hides which term binds. Which term binds is not uniform: it is the protein term for MultiSpaceNet, the untrained control and both concatenations, but the RNA term for SMART, SpaMI and SpaMosaic (their ratio column is below one). So the balance is a protein-neighbourhood score for some representations and an RNA one for others, and for those where the protein term binds it cannot fall when RNA structure is lost—which is why we describe it as a diagnostic rather than a ranking, and why the last column, the $J_{\mathrm{RNA}}/J_{\mathrm{prot}}$ ratio, is given. MultiSpaceNet trades RNA overlap for protein overlap relative to naive concatenation and so has a lower ratio, but it is not the most even representation measured: the model-free variance-scaled concatenation is. Means over the seeds available per method (as in Table S22); §three of five datasets, ‡model-free reference.

Representation	J_{RNA}	J_{prot}	Balance (min)	Ratio
**MultiSpaceNet**	0.415	0.071	0.071	5.9
MultiSpaceNet, untrained	0.185	0.037	0.037	5.0
SMART§	0.062	0.067	0.062	0.9
GraphST	0.117	0.049	0.049	2.4
STAGATE	0.052	0.043	0.043	1.2
SpaGCN	0.039	0.037	0.037	1.0
SpaMI§	0.036	0.037	0.036	1.0
SpaMosaic§	0.037	0.058	0.037	0.6
Naive concatenation	0.708	0.046	0.046	15.4
Variance-scaled concat.‡	0.224	0.181	0.181	1.2

Table S24. The same untrained-network control, applied to two published comparators. For each method the embedding is taken twice under that method's own code: once after its normal training, once from the freshly initialised network with the training loop skipped and nothing else changed — same architecture, same initialisation distribution, same preprocessing, same inputs. Both arms are scored identically: L_2 normalisation, a PCA dimension cap of 20, then `mclust`(EEE, seed 2020) at the ground-truth K. Ten seeds per arm; the two arms of a method share their seeds, so Δ is a paired quantity and is reported as mean ± SD over those ten paired differences. The quantity compared *across* methods is each method's own Δ; the untrained arms are not comparable to one another, since different architectures give different fixed feature maps. As a check on the procedure, both comparators' trained means reproduce their Table 2 values (0.298 and 0.317 against 0.299 and 0.316), so the only difference between a method's two arms is whether its optimiser ran. Across the five datasets, the descriptive mean changes are +0.179 for MultiSpaceNet, +0.054 for SMART and +0.013 for SpatialGlue; the directions are positive on 5/5, 5/5 and 3/5 datasets, respectively. We do not use a cross-architecture test or ratio as novelty evidence: the models have different objectives and parameterisations, and five datasets provide too few independent units for a well-powered non-parametric comparison. The table is retained only to show each method's own trained-versus-initialised change under its implementation.

	**MultiSpaceNet**			SMART			SpatialGlue		
Dataset	untr.	train.	Δ	untr.	train.	Δ	untr.	train.	Δ
Spleen Rep1	0.324	0.394	+0.070 ± 0.048	0.237	0.340	+0.103 ± 0.074	0.366	0.298	−0.068 ± 0.101
Spleen Rep2	0.173	0.264	+0.091 ± 0.015	0.150	0.194	+0.044 ± 0.043	0.158	0.150	−0.008 ± 0.026
Thymus Rep1	0.371	0.467	+0.096 ± 0.042	0.265	0.289	+0.024 ± 0.024	0.360	0.367	+0.007 ± 0.041
Thymus Rep2	0.406	0.707	+0.301 ± 0.101	0.324	0.393	+0.069 ± 0.024	0.441	0.513	+0.072 ± 0.052
Lymph Node A1	0.278	0.615	+0.337 ± 0.226	0.240	0.272	+0.031 ± 0.147	0.195	0.259	+0.064 ± 0.100
Mean	0.310	0.489	**+0.179**	0.243	0.298	+0.054	0.304	0.317	+0.013
Datasets improved		**5/5**			5/5			3/5	

Table S25. Local modality-corruption stress test. After fitting the model, we select a contiguous 30% spatial patch, replace the indicated modality's feature rows with rows sampled from outside that patch, rebuild the affected feature graph, and compare the original per-cell fusion with a dataset-global mean weight and fixed equal weights. Results pool five datasets and five seeds ($n = 25$ dataset–seed runs per modality; mean $\pm$ SD). Weight drop is the clean-minus-corrupted mean weight of the damaged branch within the patch; a positive value is the hypothesised response. Attention AUC measures whether the negative corrupted-branch weight ranks patch cells above non-patch cells. Neither RNA nor protein corruption is reliably localised: AUC is near or below chance and weight shifts are negligible. ARI differences are highly variable and do not establish a consistent per-cell-fusion advantage. This negative control is why the attention maps are treated as descriptive rather than causal or quality-calibrated.

Corrupted branch	Weight drop	Attention AUC	Per-cell ARI	Global ARI	Equal ARI	Per-cell wins vs. global/equal
RNA	0.003 ± 0.009	0.511 ± 0.079	0.286 ± 0.106	0.288 ± 0.124	0.307 ± 0.130	13/25; 11/25
Protein	-0.005 ± 0.005	0.420 ± 0.082	0.507 ± 0.157	0.490 ± 0.174	0.449 ± 0.151	15/25; 17/25

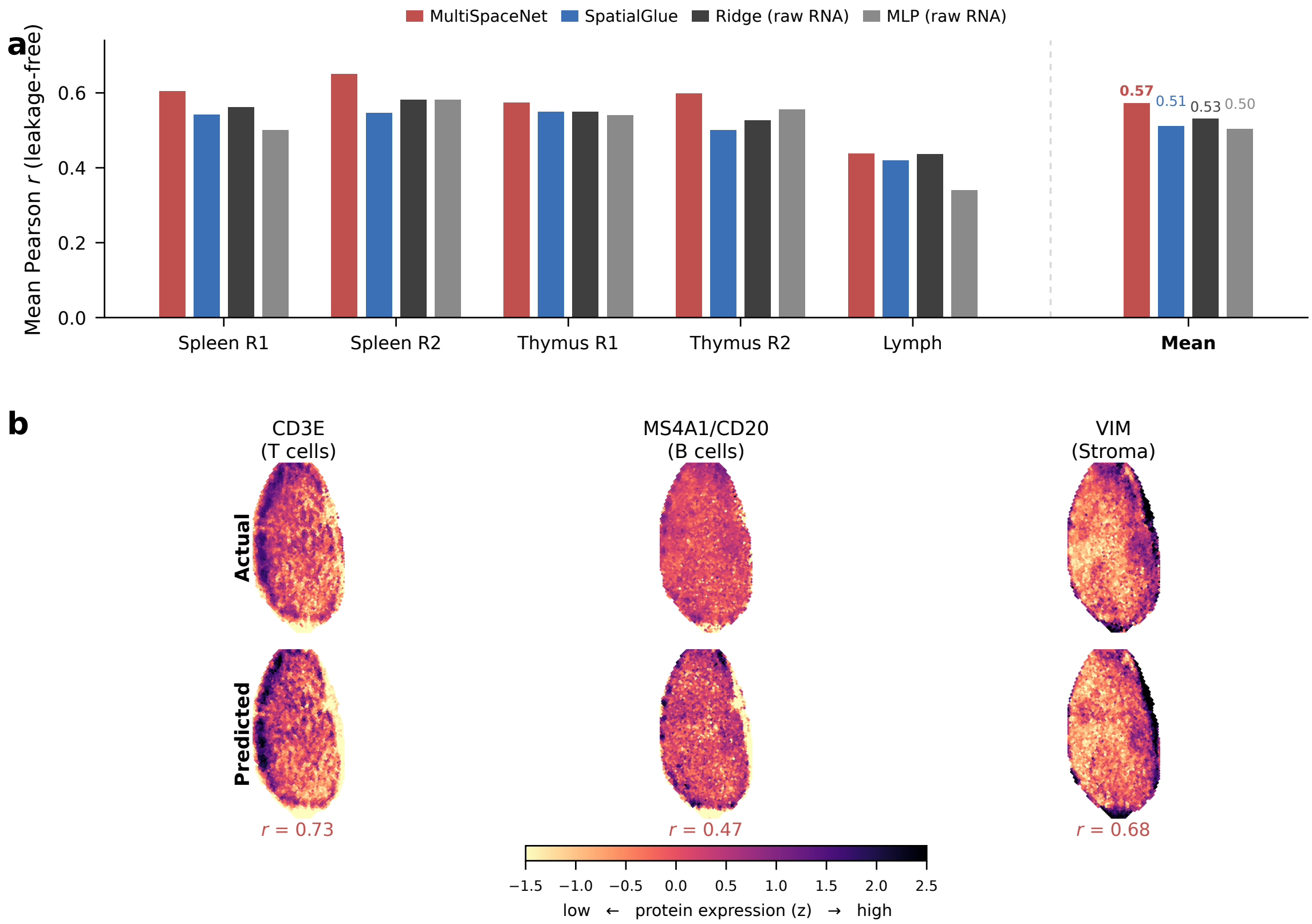

Fig. S8. Cross-modal protein expression prediction under the leakage-free protocol. **(a)** Mean Pearson r over signal-bearing proteins for MultiSpaceNet versus SpatialGlue and a Ridge and an MLP on raw RNA, across five datasets and the mean. MultiSpaceNet is highest (0.573; SpatialGlue 0.511, Ridge 0.531, MLP 0.504) and wins on 98/110 signal-bearing targets versus SpatialGlue. The panel is reported as a practical imputation comparison rather than as a ranking of representations. The generative imputers totalVI and sciPENN are compared, with their normalisation caveat, in Supplementary Table S15. **(b)** Actual (top) and predicted (bottom) spatial expression for three representative markers on the Human Lymph Node dataset: CD3E (T cell), MS4A1/CD20 (B cell), and VIM (stroma). Predicted maps recapitulate the spatial expression patterns; the leakage-free Pearson r for each marker is shown beneath its panel.

References

1. Lawrence Hubert and Phipps Arabie. Comparing partitions. *Journal of Classification*, 2(1):193–218, 1985. doi: 10.1007/BF01908075.
2. Giovanni Palla, Hannah Spitzer, Michal Klein, David Fischer, Anna Christina Schaar, Louis Benedikt Kuemmerle, Sergei Rybakov, Ignacio L Ibarra, Olle Holmberg, Isaac Virshup, et al. Squidpy: a scalable framework for spatial omics analysis. *Nature Methods*, 19(2):171–178, 2022. doi: 10.1038/s41592-021-01358-2.
3. Xiao Wang, Houye Ji, Chuan Shi, Bai Wang, Yanfang Ye, Peng Cui, and Philip S. Yu. Heterogeneous graph attention network. In *The World Wide Web Conference (WWW)*, pages 2022–2032, 2019. doi: 10.1145/3308558.3313562.
4. William L. Hamilton, Rex Ying, and Jure Leskovec. Inductive representation learning on large graphs. In *Advances in Neural Information Processing Systems (NeurIPS)*, volume 30, pages 1024–1034, 2017.
5. Xiangxiang Wang, Yanchi Su, Gaoyang Hao, Meng Wang, Yunhe Wang, and Xiangtao Li. ARISE: Rna-anchored shared-edge topology and hierarchical fusion for spatial multi-omics integration. *Bioinformatics*, 42(7):btag465, 2026. doi: 10.1093/bioinformatics/btag465. URL `https://doi.org/10.1093/bioinformatics/btag465`.
